# A TAF10‐ERF109 Transcriptional Module Directs Flavonoid‐Based Stress Resilience and Yield Enhancement in Foxtail Millet and Wheat

**DOI:** 10.1111/pbi.70711

**Published:** 2026-06-26

**Authors:** Meng Zhang, Kangwei Wang, Jiayin Fan, Yue Zheng, Qi Zhang, Haoyang Du, Shenghui Xiao, Jinguang Huang, Kang Yan, Shizhong Zhang, Qiang He, Guanqing Jia, Chengchao Zheng, Xianmin Diao, Guodong Yang, Changai Wu

**Affiliations:** ^1^ State Key Laboratory of Wheat Improvement Shandong Agricultural University Tai'an China; ^2^ Institute of Crop Sciences Chinese Academy of Agricultural Sciences Beijing China; ^3^ National Center of Technology Innovation for Comprehensive Utilization of Saline‐Alkali Land Engineering Center of Saline‐Alkali Soil Plant ‐ Microbial Joint Restoration Dongying China

**Keywords:** flavonoids, foxtail millet ( *Setaria italica* ), salt and drought stress, SiF3'H, SiTAF10‐SiERF109 module

## Abstract

To ensure sustainable agricultural production under escalating environmental constraints such as drought and salinity, innovative strategies are urgently needed. Here, we reveal in foxtail millet ( *Setaria italica* ) that transcription factors TAF10 and ERF109 form a functional complex which co‐activates the expression of key flavonoid biosynthesis genes (*SiPAL*, *SiF3′H*, *SiFLS*, *SiF3H*), thereby enhancing abiotic stress tolerance. Application of specific flavonoids (apigenin, naringenin, hesperetin) rescues stress‐sensitive phenotypes in *ERF109‐RNAi* and *taf10* lines. Importantly, field‐based application protocols for apigenin were established in both foxtail millet and wheat ( *Triticum aestivum* ), leading to yield increases of 12.3%–19.6% in wheat and 15.8%–23.9% in foxtail millet under drought and saline field conditions. Our study not only delineates a conserved transcriptional mechanism regulating flavonoid‐mediated stress resilience but also delivers a translatable, eco‐friendly biostimulant strategy to enhance crop productivity in stress‐affected environments.

## 1 Introduction

Abiotic stress, including drought and salinity stress, represent major constraints to global crop productivity and pose severe threats to food security (Zhang et al. 2022). Projections indicate that by 2050, these two stress factors alone will be responsible for a worldwide drop in crop yields of 10%–50%, with especially devastating consequences for agriculture in vulnerable regions (FAO 2021). Although genetic engineering and breeding for improved stress resilience are active areas of investigation, there is a growing demand for sustainable, non‐genetic strategies that can be rapidly deployed. Plant biostimulants offer such a promising avenue. For example, application of biostimulant steroids extracted from soybean ( *Glycine max* ), including fucosterol and soyasaponin II, significantly improve the drought tolerance of seedlings from soybean, wheat ( *Triticum aestivum* ), foxtail millet ( *Setaria italica* ) and maize ( *Zea mays* ) under laboratory conditions (Yu et al. 2024). Yield of spring wheat Cadenza is increased up to 18% when trehalose 6‐phosphate (T6P) spray is applied during the grain filling period (Griffiths et al. 2016). Under both well‐watered and water‐stressed conditions over 4 years, the plant‐permeable, sunlight‐activated T6P signalling precursor DMNB‐T6P stimulated an average yield increase 10.4% yield across three elite wheat varieties (Griffiths et al. 2025). Grain yield of maize, rice or rapeseed is increased up to 9.8% when thiamine pyrophosphate (TPP) spray is applied (Luo et al. 2026). Despite these promising results, the broader development of biostimulants is often hindered by an insufficient mechanistic understanding of their biosynthesis and their regulation, as well as a notable lack of systematic reporting on application protocols and their effects on crop yield, particularly under natural abiotic stress conditions such as drought and salinity.

Flavonoids, a widespread class of polyphenolic compounds, play multifaceted roles in plant stress acclimation. They act as antioxidants that scavenge reactive oxygen species (ROS), serve as chemoattractants facilitating rhizobial and mycorrhizal associations to improve nitrogen, iron and phosphorus acquisition and regulate auxin transport to modulate growth and yield under normal and stress conditions (Chapman and Muday 2021; Kumar et al. 2023; Wu et al. 2025). These attributes make flavonoids compelling candidates as biostimulants. Supporting this notion, quercetin helps support ROS homeostasis and enhances physiological reactions under environmental stresses (Singh et al. 2021). Our previous study showed that application of apigenin (AP) could significantly improve salinity tolerance of foxtail millet seedlings (Xiao et al. 2023). Nevertheless, the practical deployment of flavonoids as topical treatments remains underexplored.

The flavonoid biosynthesis pathway is well‐characterized, involving enzymes such as phenylalanine ammonia‐lyase (PAL), chalcone synthase (CHS) and flavonoid 3′‐hydroxylase (F3′H) (Daryanavard et al. 2023). Stress‐inducible expression of genes like *F3′H* and *FLS* enhances antioxidant capacity and tolerance in various plant species (Cui et al. 2014; Gharibi et al. 2019; Ma et al. 2024; Pyrzynska 2022). Transcriptional regulation of this pathway is primarily attributed to MYB/bHLH/WD40 complexes (Feller et al. 2011; Naik et al. 2022), though recent evidence implicates ETHYLENE‐RESPONSE FACTOR (ERF)‐type family transcription factors in some species, including tomato ( *Solanum lycopersicum* ), citrus ( *Citrus sinensis* ) and Tartary buckwheat ( *Fagopyrum tataricum* ) (Ding et al. 2022; Li et al. 2020; Wan et al. 2023). The general mechanisms coordinating stress signalling with flavonoid pathway activation are not fully resolved.

In eukaryotes, the initiation of transcription depends on the assembly of general transcription factors and the transcription factor IID (TFIID) complex, which consists of the TATA‐binding protein (TBP) and TBP‐associated factors (TAFs). TAFs are essential for promoter recognition, assembly of the transcription machinery and chromatin modulation (Uffenbeck and Krebs 2006). Highly conserved from yeast ( *Saccharomyces cerevisiae* ) to humans, TAFs regulate cell differentiation, the cell cycle, organ development and stress acclimation (Basehoar et al. 2004; Bhuiyan and Timmers 2019; Ruppert et al. 1993; Uffenbeck and Krebs 2006). Recent studies highlight their importance in plant development and stress responses as well. In Arabidopsis, for instance, loss of *TAF1* function impairs pollen tube development, indicating a key role in gamete viability (Waterworth et al. 2015), while the *taf6* mutant shows defective male gametophyte transmission (Lago et al. 2005). The *taf13* mutant exhibits embryo arrest and endosperm over‐proliferation (Lindner et al. 2013). TAF15b affects flowering time through transcriptional repression of FLOWERING LOCUS C (FLC) (Eom et al. 2018). Knockdown of *TAF10* leads to abnormal meristem activity, leaf development and greater sensitivity to osmotic stress (Furumoto et al. 2005; Gao et al. 2006). These findings position TAFs as central nodes in transcriptional networks that integrate developmental and environmental signals, making them attractive targets for improving stress resilience in crops. Still, the regulatory mechanisms governing TAF function remain poorly understood.

Foxtail millet, one of the world's oldest crops with a domestication history spanning over 8000 years, has exceptional abiotic stress tolerance (Diao et al. 2014). It also possesses high nutritional value, being rich in essential amino acids, dietary fibre, B vitamins and minerals such as iron and zinc, making it an ideal crop for enhancing both food nutrition and human health (Li et al. 2022). However, the genetic and molecular bases of these traits remain largely unknown, limiting the cultivation and extension of foxtail millet as a major cereal crop and for improving overall climate resilience. In this study, we integrated genetic screening, molecular characterization, transcriptomics and metabolomics to elucidate the regulatory architecture of stress‐induced flavonoid biosynthesis. We then applied this knowledge directly to develop and field‐test a highly effective, flavonoid‐based biostimulant strategy in wheat and foxtail millet.

## 2 Results

### 2.1 Natural Variation in *F3′H* Affects Stress Tolerance and Flavonoid Accumulation

**FIGURE 1:**
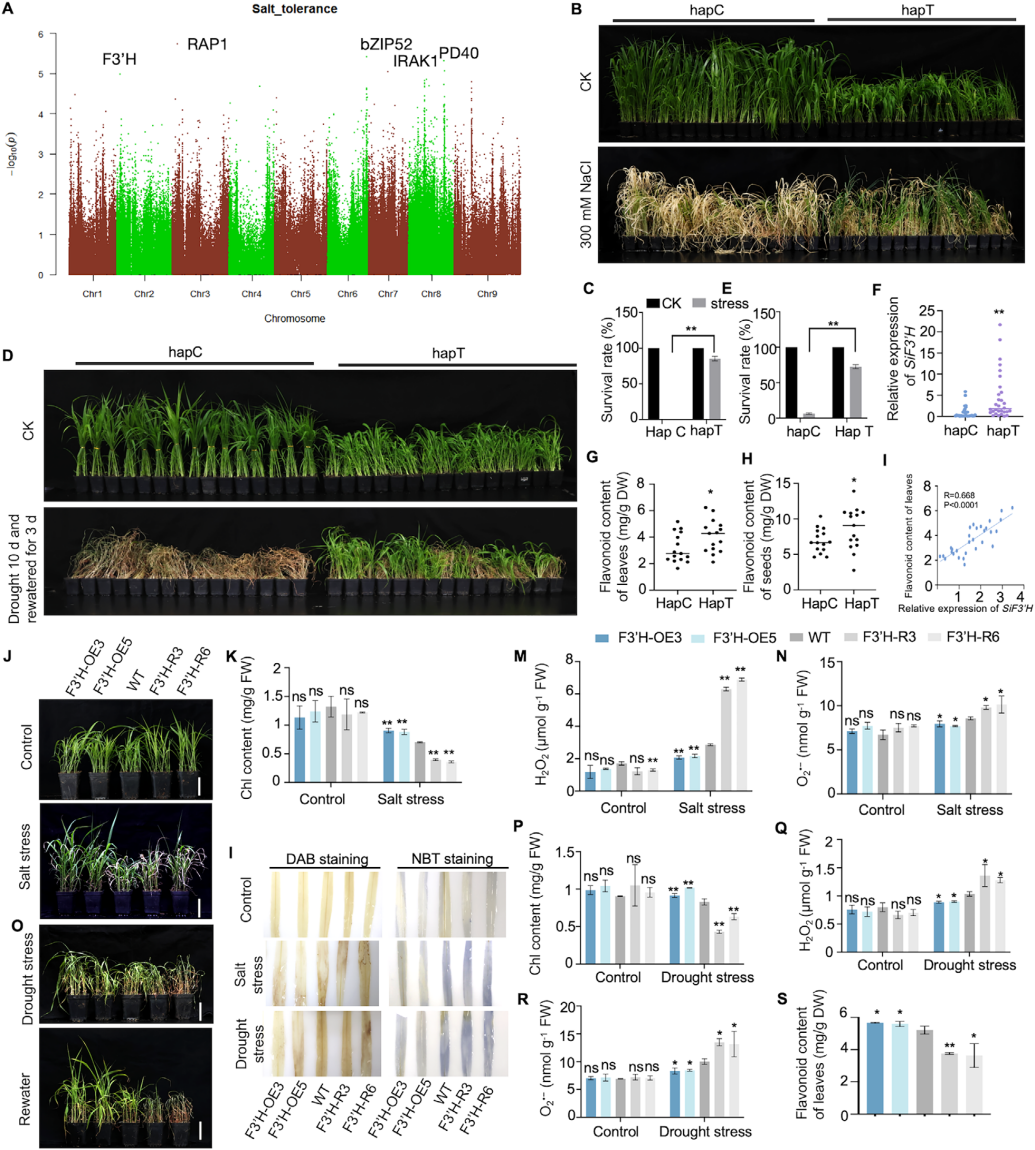
Natural variation in *SiF3′H* is associated with salt and drought resistance in foxtail millet. (A) Genome‐wide association study (GWAS) for salt tolerance of foxtail millets. The dashed horizontal line indicates the genome‐wide significance threshold (*p* ≥ 1.0 × 10^−5^). (B) Phenotypes of 15 accessions each of *SiF3′H* ^hapC^ and *SiF3′H* ^hapT^ after 10 days of treatment with 300 mM NaCl or under control conditions. (C) Survival rates of the accessions shown in (B). Data are mean ± SD; *n* = 3 biological replicates. (D) Phenotypes of *SiF3′H* ^hapC^ and *SiF3′H* ^hapT^ accessions following 30 days of drought stress and 3 days of rewatering. (E) Survival rates of plants shown in (D). Data are mean ± SD; *n* = 3. (F) Relative expression of *SiF3′H* in two haplotypes, detected by RT‐qPCR. (G and H) Flavonoid content in leaves (G) and seeds (H) of *SiF3′H* ^hapC^ and *SiF3′H* ^hapT^ plants. (I) Correlation between *SiF3′H* expression and leaf flavonoid content across haplotypes. (J and O) Phenotypes of WT and transgenic seedlings under control conditions, 300 mM NaCl for 10 days (J) or after 30 days of drought followed by 3 days of rewatering (I). Scale bars = 10 cm. (K and P) chlorophyll (Chl) content of plants from (J) and (O). Data are mean ± SD; *n* = 3. (L) NBT and DAB staining of leaves from WT and transgenic plants under control, salt or drought stress. (M, N, Q and R) H_2_O_2_ and $O_{2}^{˙-}$ levels in leaves under corresponding treatments. (S) Flavonoid contents in leaves of WT and transgenic plants. Unless otherwise noted, data are mean ± SD of three biological replicates. Significance was assessed by Student's *t*‐test: *p < 0.05, **p < 0.01; ns, not significant.

To elucidate the genetic bases of abiotic stress tolerance in foxtail millet, a genome‐wide association study (GWAS) for salinity tolerance was conducted across 731 foxtail millet accessions. This study identified a significant association with *SiF3′H*, encoding flavonoid 3′‐hydroxylase, a key enzyme in the flavonoid biosynthetic pathway (Figure 1A and Table S1). A single C/T polymorphism at position −845 bp relative to the ATG defined two major haplotypes, named here *HapC* and *HapT*. Accessions carrying *HapT* exhibited markedly enhanced salinity tolerance, with an 80% survival rate, whereas accessions harbouring *HapC* all died under saline conditions (Figure 1B,C). This superior tolerance extended to drought stress, under which accessions with *HapT* showed a significantly higher survival rate than those with *HapC* (Figure 1D,E). Consistent with these observations, *SiF3′H* transcript levels were 2.1‐fold higher in accessions with the *HapT* haplotype than in those with *HapC* (Figure 1F) and accessions carrying *HapT* accumulated twice as much flavonoid in their leaves and seeds as accessions harbouring *HapC* (Figure 1G,H). We observed a strong positive correlation (*R* = 0.67) between *SiF3′H* expression levels and flavonoid content (Figure 1I), suggesting that natural variation in *SiF3′H* expression contributes to stress acclimation via flavonoid accumulation.

To functionally validate this relationship, transgenic lines in the ‘xiaomi’ genetic background (wild type, WT) were generated. Overexpression lines (*F3′H*‐OE) showed 4‐ to 12‐fold higher *SiF3′H* transcript levels, while artificial microRNA‐mediated knockdown lines (*F3′H*‐R) exhibited 20%–70% lower *F3′H* transcript levels (Figure S1). Under individual salinity or drought stress, *F3′H‐*OE seedlings displayed robust growth and elevated chlorophyll contents, whereas *F3′H‐*R plants showed severe wilting and lower chlorophyll accumulation, all relative to the WT (Figure 1J,K,O,P). The growth phenotypes of *F3′H‐*OE plants were associated with 50%–60% lower H_2_O_2_ and $O_{2}^{˙-}$ levels under both salinity and drought stress relative to normal growth conditions (Figure 1L–NQ,R), alongside a 50% higher total flavonoid content relative to that of WT plants (Figure 1S). By contrast, *F3′H‐*R plants exhibited diminished flavonoid accumulation. These results suggest that *SiF3′H* enhances abiotic stress tolerance through flavonoid‐mediated ROS scavenging.

### 2.2 ERF109 Directly Activates *F3′H* Expression in Response to Stress

A time‐course expression analysis revealed the rapid induction of *SiF3′H* in the shoots (but not roots) of seedlings within 3 h of treatment with salinity or drought stress (Figure S2A,B). To identify upstream regulators of *SiF3′H* transcription, its promoter was analysed and a dehydration‐responsive element (DRE) core motif was identified (Figure S3). A subset of 20 *SiERF*, the candidate DRE binding transcription factor, was selected from the total 167 members of this family to detect the stress responsive expression (Figure S4). *SiERF109* was the only gene with significant induction under both salinity and drought and it was co‐upregulated with *SiF3′H* (Figure S5A–D). Tissue‐specific expression patterns also partially overlapped with *SiF3′H* (Figure S5E), suggesting a potential regulatory relationship.

**FIGURE 2:**
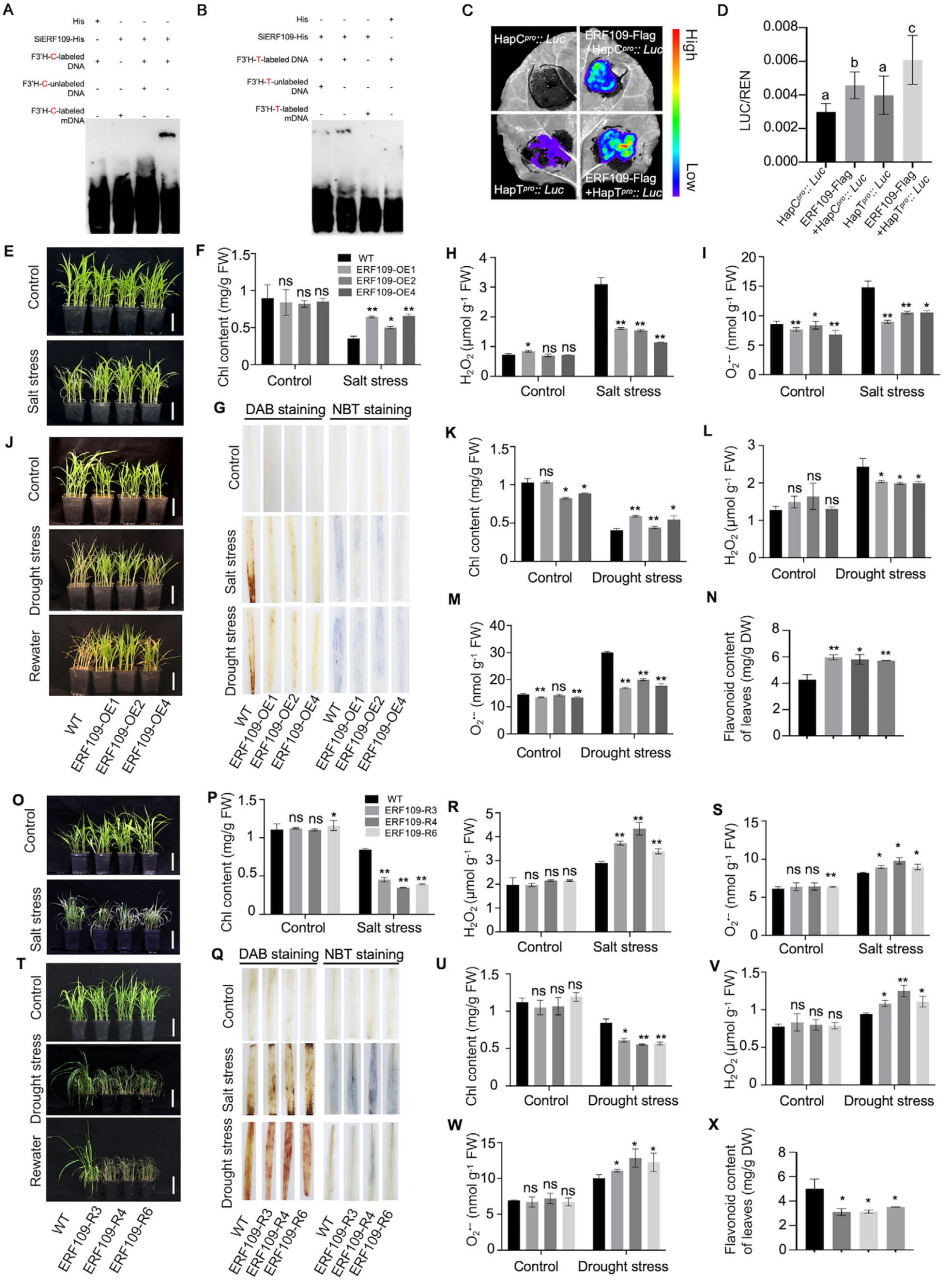
SiERF109 directly activates SiF3*′*H in response to stress. (A and B) Electrophoretic mobility shift assay (EMSA) using biotin‐labelled probes containing the DRE core element (5′‐GGG/ACCG‐3′) from *SiF3′H* ^hapC^ and *SiF3′H* ^hapT^ promoters. Unlabeled wild‐type probes served as competitors, a mutant probe (5′‐AAA/AAAA‐3′) served as a negative control. (C) Luciferase (LUC) reporter assay showing SiERF109‐mediated transactivation of the *F3′H* ^HapC^ and *F3′H* ^HapT^promoters. Data are mean ± SD (*n* = 3 biological replicates). (D) Normalized LUC/REN activity from assays in (C), with 35S:REN as an internal control. Data are mean ± SD; *n* = 3. (E–M) Phenotypes (E and J), Chlorophyll (Chl) contents (F and K), NBT and DAB staining (G), H_2_O_2_ and $O_{2}^{˙-}$ levels (H, I, L, M) in WT and *SiERF109*‐overexpression (*SiERF109*‐OE) seedlings under control conditions, 300 mM NaCl for 10 days or after 30 days of drought followed by 3 days of rewatering. Scale bars = 10 cm. Data are mean ± SD (*n* = 3). (N) Flavonoid contents in leaves of WT and *ERF109*‐OE plants. Data are mean ± SD (*n* = 3). (O–W) Phenotypes (O and T), Chlorophyll (Chl) contents (P and U), NBT and DAB staining (Q), H_2_O_2_ and $O_{2}^{˙-}$ levels (R, S, V and W) in WT and *SiERF109*‐RNAi interference (*ERF109*‐R) seedlings under the same treatments as above. Scale bars = 10 cm. Data are mean ± SD (*n* = 3). (X) Flavonoid contents in leaves of WT and *ERF109*‐R plants. Data are mean ± SD (*n* = 3). Statistical significance of differences was determined using Student's *t*‐test (*p < 0.05 and **p < 0.01).

To test this regulatory relationship, we performed electrophoretic mobility shift assays (EMSAs), which demonstrated that recombinant purified SiERF109‐His directly binds to the DRE motif in the promoter of *SiF3′H* from both haplotypes (Figure 2A,B). Luciferase reporter assays in the leaves of *Nicotiana benthamiana* plants further showed that SiERF109 activates transcription from the *SiF3′H* promoter, with stronger transactivation of the *HapT* promoter variant (Figure 2C,D).

Genetic evidence supported this regulation. Indeed, *SiERF109‐*OE lines (3.6‐ to 26‐fold overexpression; Figure S6A) exhibited enhanced salinity stress tolerance, displaying on average 40% higher chlorophyll accumulation than the WT under the same conditions (Figure 2E,F). Consistent with a role in oxidative stress mitigation, *SiERF109‐*OE plants accumulated 30%–60% less H_2_O_2_ and $O_{2}^{˙-}$, as indicated by staining with diaminobenzidine (DAB) and nitroblue tetrazolium (NBT) and quantified by biochemical assays (Figure 2G–I). These differences were associated with a threefold to fivefold upregulation of *SiF3′H* transcript levels (Figure S7A) and a 30% greater accumulation of total flavonoids in these *SiERF109‐*OE lines relative to the WT (Figure 2N). Conversely, RNA interference (RNAi)‐mediated knockdown lines (*SiERF109‐*R, 30%–60% lower transcript levels) showed heightened stress sensitivity, severely lower chlorophyll contents (Figure S6B and Figure 2O,P), 30%–40% more ROS (Figure 2Q–S), 60%–75% lower *SiF3′H* expression (Figure S7B) and 40% lower flavonoid levels (Figure 2X). Tests under drought stress (Figure 2J–N,T–X) confirmed that *SiERF109* is a central regulator of flavonoid‐mediated ROS homeostasis across multiple abiotic stresses.

### 2.3 TAF10 Interacts With ERF109 to Co‐Activate *F3′H* Transcription

**FIGURE 3:**
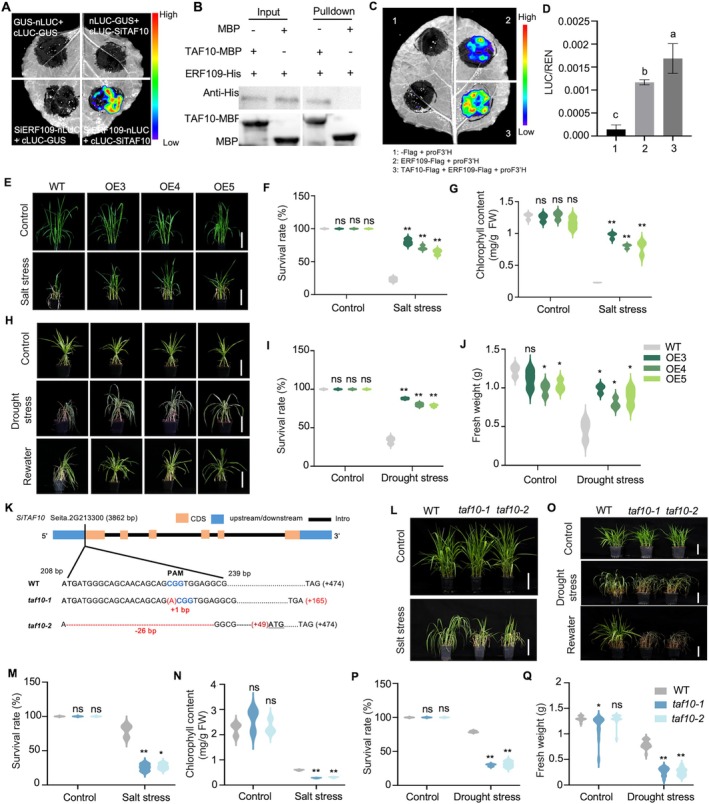
SiTAF10 interacts with SiERF109 to regulate stress responses. (A) Luciferase complementation imaging assay showing in vivo interaction between SiTAF10 and SiERF109. Negative controls included nLUC + cLUC, SiERF109‐nLUC + cLUC and nLUC + SiTAF10‐cLUC combinations. Data represent mean ± SD (*n* = 3 biological replicates). (B) In vitro pull‐down assay confirming direct interaction between recombinant SiTAF10 and SiERF109 proteins. (C and D) Functional dissection of SiERF109‐mediated *SiF3′H* promoter activation by SiTAF10. Numbers indicate tested combinations. (E) Phenotypes of WT and *SiTAF10* transgenic seedlings under control conditions or 300 mM NaCl treatment for 10 days. Scale bars = 10 cm. (F and G) Survival rates (F) and chlorophyll contents (G) of plants shown in (E). Data represent mean ± SD (*n* = 3). (H) Phenotypes of WT and *SiTAF10* transgenic plants following 30 days of drought and 3 days of rewatering. Scale bars = 10 cm. (I and J) Survival rates (I) and fresh weight (J) after the drought treatment. Data represent mean ± SD (*n* = 3). (K) Sequencing chromatograms of CRISPR/Cas9‐induced *taf10* mutant alleles. Edited sites are highlighted in red; PAM sequences (NGG) are shown in blue. (L) Phenotypes of *taf10* mutants and WT plants after 10 days of NaCl treatment. Scale bars = 10 cm. (M and N) Survival rates (M) and chlorophyll contents (N) of *taf10* mutants and WT seedlings following salt stress. Data represent mean ± SD (*n* = 3). (O) Phenotypes of *taf10* mutants and WT seedlings after 30 days of drought and 3 days of rewatering. Scale bars = 10 cm. (P and Q) Survival rates (P), and fresh weight (Q) of *taf10* mutants and WT plants after drought. Data represent mean ± SD (*n* = 3). Statistical significance was determined by Student's *t*‐test (*p < 0.05, **p < 0.01; ns, not significant).

To further dissect the regulatory mechanism of *SiF3′H* transcription, a yeast two‐hybrid screen using the C‐terminal domain of SiERF109 as bait against a cDNA library prepared from total RNA extracted from young seedlings was conducted. SiTAF10, a component of the TFIID complex, was identified as a potential interactor of SiERF109 (Table S2). This physical interaction was confirmed through luciferase complementation imaging assays (LCAs), pull‐down experiments and bimolecular fluorescence complementation (BiFC) assays (Figure 3A,B and Figure S8). Luciferase reporter assays further demonstrated that SiTAF10 enhances SiERF109‐mediated transactivation of *SiF3′H* (Figure 3C,D).

Mirroring the kinetics of *SiERF109* induction (Figure S5A–D), *SiTAF10* expression was rapidly induced in both shoots and roots of foxtail millet seedlings within 3 h of salt or drought treatment (Figure S9A–D), with several overlaps in different tissues (Figures S5E and S9E), suggesting functional cooperation.

To determine the function of *SiTAF10*, transgenic lines were generated. *SiTAF10* overexpression (*TAF10*‐OE) lines with 6‐ to 25‐fold overexpression were constructed (Figure S9F). They displayed enhanced tolerance to salinity stress, with better growth, higher survival rates and more chlorophyll than the WT (Figure 3E–G). Similar benefits under drought stress were observed (Figure 3H–J). Conversely, two *taf10* mutants, generated via clustered regularly interspaced short palindromic repeats (CRISPR)/CRISPR‐associated nuclease 9 (Cas9)‐mediated editing, were hypersensitive to stress (Figure 3K–Q; Figure S9G–I) and accumulated more ROS (Figure S10). Changes in flavonoid levels were associated with corresponding changes in *SiF3′H* expression levels in *SiTAF10*‐OE and *taf10* lines (Figures 1S and 2N,X, Figure S11), confirming that the TAF10–ERF109 module coregulates *SiF3H* expression and flavonoid‐dependent ROS homeostasis under multiple stress conditions.

### 2.4 The TAF10 **–** ERF109 Module Regulates the Entire Flavonoid Pathway

**FIGURE 4:**
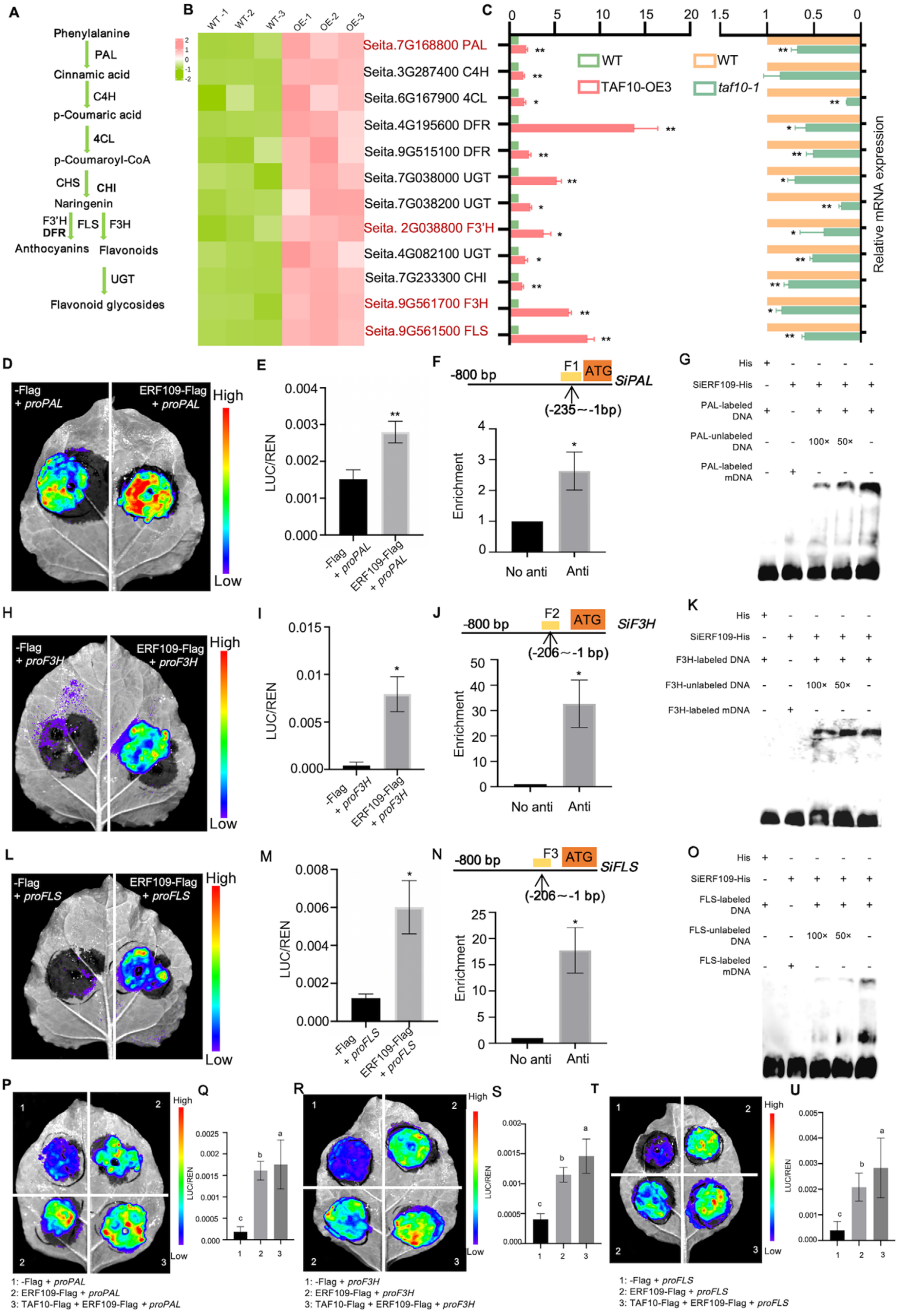
SiERF109 and SiTAF10 co‐activate *SiPAL*, *SiF3H*, *SiFLS* and *SiF3′H*. (A) Schematic of the flavonoid biosynthesis pathway in foxtail millet, highlighting key enzymes examined. (B) Heatmap of RNA‐seq expression profiles for flavonoid pathway genes in WT and *SiTAF10*‐OE seedlings. (C) RT‐qPCR validation of transcript levels of *PAL*, *C4H*, *4CL*, *DFRs*, *UGTs*, *F3′H*, *FLS*, *CHI* and *F3H* in *SiTAF10*‐OE and *taf10* mutant seedlings. (D, H and L) Luciferase reporter assays in *N. benthamiana* leaves co‐infiltrated with Agrobacterium carrying SiERF109‐FLAG or an empty FLAG vector, together with the reporter constructs SiPALpro (−2000 to −1)::LUC (D), SiF3Hpro (−2000 to −1)::LUC (H) and SiFLSpro (−2000 to −1)::LUC (L), Images were taken 48 h after infiltration following luciferin application. (E, I and M) Quantification of dual‐luciferase assays showing SiERF109‐mediated activation of the *SiPAL* (E), *SiF3H* (I) and *SiFLS* (M) promoters. Activity was normalized to the 35S:REN internal control (LUC/REN ratio). Data represent mean ± SD (*n* = 3 biological replicates). **p < 0.01 (Student's *t*‐test). (F, J and N) ChIP‐qPCR analysis of SiERF109 binding to promoter regions (F1–F3) containing GC‐box motifs in *SiPAL* (F), *SiF3H* (J) and *SiFLS* (N). Data represent mean ± SD (*n* = 3). *p < 0.05, **p < 0.01 (Student's *t*‐test). (G, K and O) EMSA validation of SiERF109 binding to the GC‐box (GCCGCC) motif in *SiPAL* (G), *SiF3H* (K) and *SiFLS* (O). Assays included biotin‐labelled wild‐type probes (labelled DNA), unlabeled competitors (unlabeled DNA) and mutant probes (labelled mDNA, AAAAAA). (P–U) Functional analysis of SiTAF10‐dependent enhancement of SiERF109‐mediated trans‐activation of *SiPAL* (P and Q), *SiF3H* (R and S) and *SiFLS* (T and U). Numbers indicate the experimental combinations.

To understand the global influence of SiTAF10 on gene expression, transcriptome deep sequencing (RNA‐seq) of the WT and *SiTAF10‐*OE plants was performed. This comprehensive analysis identified 6110 differentially expressed genes (DEGs), with significant enrichment in secondary metabolite biosynthesis pathways (Figure S12). Of particular interest, 22 flavonoid biosynthesis genes were upregulated, including *SiPAL*, *SiF3H*, *SiFLS* and *SiF3′H* (Figure 4A–C). Chromatin immunoprecipitation quantitative PCR (ChIP‐qPCR) assays confirmed that SiTAF10 is associated with the *SiPAL*, *SiF3H*, *SiFLS* and *SiF3′H* promoters (Figure S13). The expression of these genes was all induced by salinity or drought stress treatment similarly to that of *SiTAF10* and *SiERF109*, as well as in some overlapping tissues (Figure S14). However, SiTAF10 alone neither activated these promoters in luciferase reporter assays nor bound directly in EMSAs (Figure S15), suggesting it acts as a cofactor rather than a direct transcriptional activator that directly binds to DNA.

A closer look at the promoters of *SiPAL*, *SiF3H* and *SiFLS* identified DRE core elements; moreover, the expression of these genes was upregulated in *SiERF109*‐OE lines while inhibited in *SiERF109‐R* lines relative to the WT (Figure S16A–D). Luciferase reporter assays, ChIP‐qPCR and EMSAs confirmed that SiERF109 binds directly to the DRE motifs in these promoters (Figure 4D–O). Importantly, SiTAF10 enhanced the SiERF109‐mediated transcriptional transactivation of all three genes (Figure 4P–U). However, the target activation effect of SiERF109 on *SiPAL*, *SiF3H*, *SiFLS* and *SiF3′H* disappeared in the *SiTAF10* knockout background (Figure S16E). These results established a non‐canonical TAF10‐dependent co‐activation module for flavonoid biosynthesis genes, distinct from the canonical MBW complex.

**FIGURE 5:**
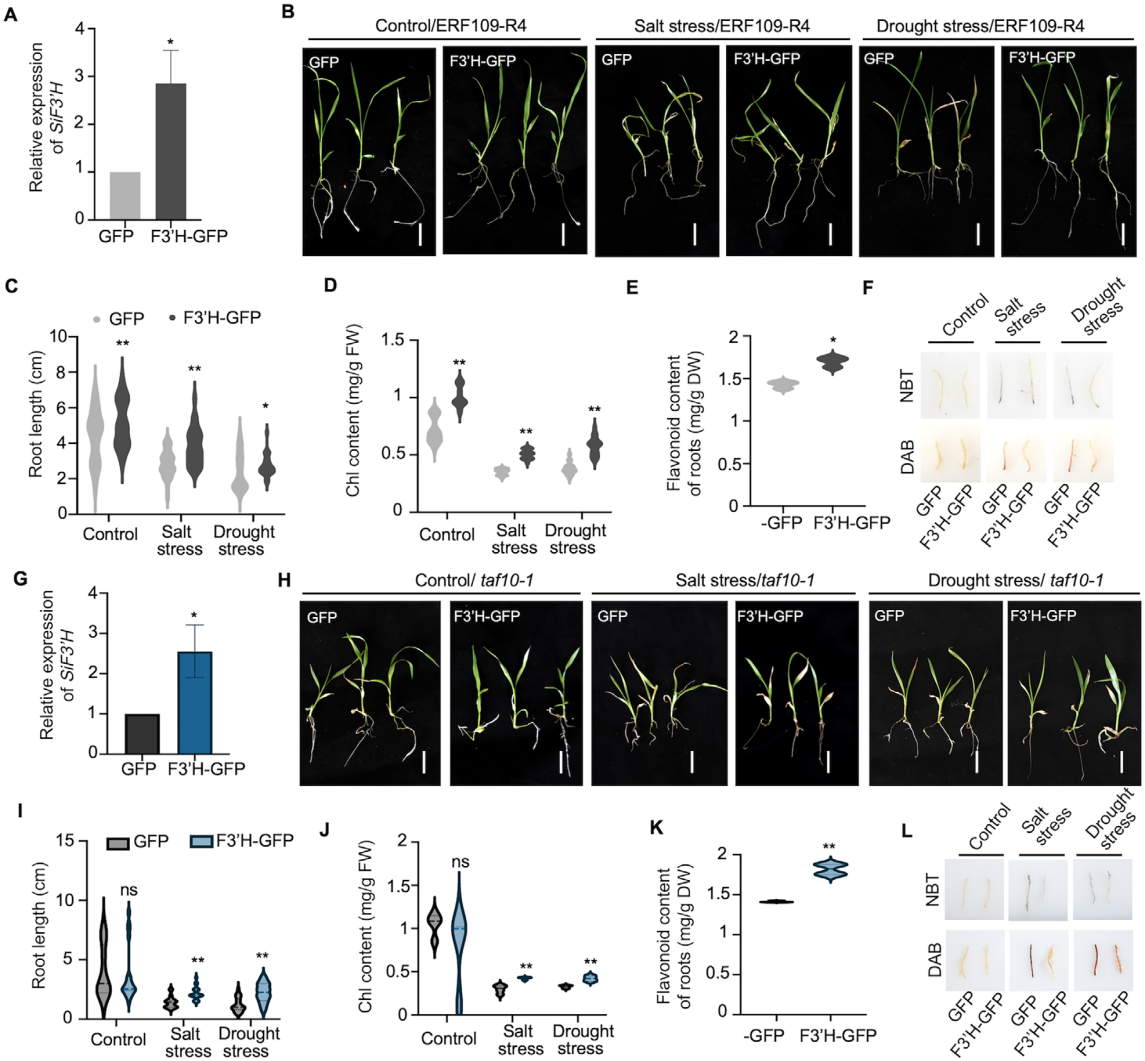
SiF3*′*H rescues salt and drought sensitivity of *SiERF109*‐R and *taf10* mutant seedlings through flavonoids‐mediated ROS scavenging. (A and G) Transcript levels of *SiF3′H* in control (GFP) and SiF3*′*H‐overexpressing (F3*′*H‐GFP) hairy roots in the *SiERF109*‐R (A) and *taf10* mutant (A) backgrounds. Data represent mean ± SD (*n* = 3). (B and H) Phenotypic rescue of *SiERF109*‐R (B) and *taf10* mutant (H) seedlings by SiF3*′*H‐GFP hairy roots under control, 300 mM NaCl or drought conditions. Scale bars = 2 cm. (C, D, I, J) Quantitative analysis of stress responses shown in (B and H), based on primary root length (C and I) and chlorophyll (Chl) content (D and J). (E and K) Total flavonoid accumulation in control and F3'H‐GFP transgenic hairy roots of *SiERF109*‐R (E) and *taf10* mutant (K) lines. (F and L) Detection of ROS in hairy roots by NBT staining ($O_{2}^{˙-}$) and DAB staining (H_2_O_2_) under control and stress conditions. Statistical significance: *p < 0.05, **p < 0.01; ns, not significant (Student's *t*‐test). All experiments included three biological replicates.

To validate the functional significance of *SiF3′H* and *SiPAL* expression on flavonoid content, a genetic complementation assay by expressing *SiF3′H‐GFP* (encoding a fusion of SiF3′H with the green fluorescent protein) in *ERF109‐*R4 hairy roots was performed (Figure 5A,G). Under stress, *SiF3′H‐GFP* transgenic seedlings showed improved growth, 35% longer roots, 30%–45% higher chlorophyll content, 15% more flavonoids (Figure 5B–E) and obviously lower ROS levels (Figure 5F). We observed a similar phenotypic rescue in the *taf10‐1* mutant expressing *SiF3′H‐GFP* (Figure 5H–L). Expressing *SiPAL‐GFP* also restored the stress tolerance of *ERF109‐*R4 and *taf10‐1* seedlings back to levels of the WT (Figure S17), confirming that *SiF3′H* and *SiPAL* are key genes downstream of the ERF109–TAF10 module.

### 2.5 Metabolic Output of the ERF109 **–** TAF10 Module Rescues Their Genetic Defects at the Seedling Stage

To directly test how many types of flavonoids were affected by the ERF109–TAF10 module, we performed metabolomic profiling of 60‐day‐old *SiTAF10*‐OE and the WT plants under normal, salinity stress and drought stress conditions, which identified 1729 metabolites (Table S3). A principal component analysis (PCA) revealed stress‐specific reprogramming of the metabolome (Figure S18A), with 554 significantly differentially abundant metabolites (SDMs; variable importance in projection [VIP] ≥ 1, |fold‐change| ≥ 1) (Figure S18B–E). Flavonoids constituted 13.7% of all SDMs and showed stress‐dependent accumulation: exposure to drought stress lowered the levels of six flavones and three flavonols, while salinity stress raised the contents of one chalcone, three flavanones, nine flavones and 15 flavonols in *SiTAF10*‐OE plants compared with the WT (Figure S19). Seeds from *SiTAF10‐OE*, *SiERF109‐OE*, *SiF3′H‐OE* lines also had elevated flavonoid contents, whereas their RNAi lines showed lower contents (Figure S20), supporting a role for flavonoids in enhanced antioxidant capacity and beneficial value.

**FIGURE 6:**
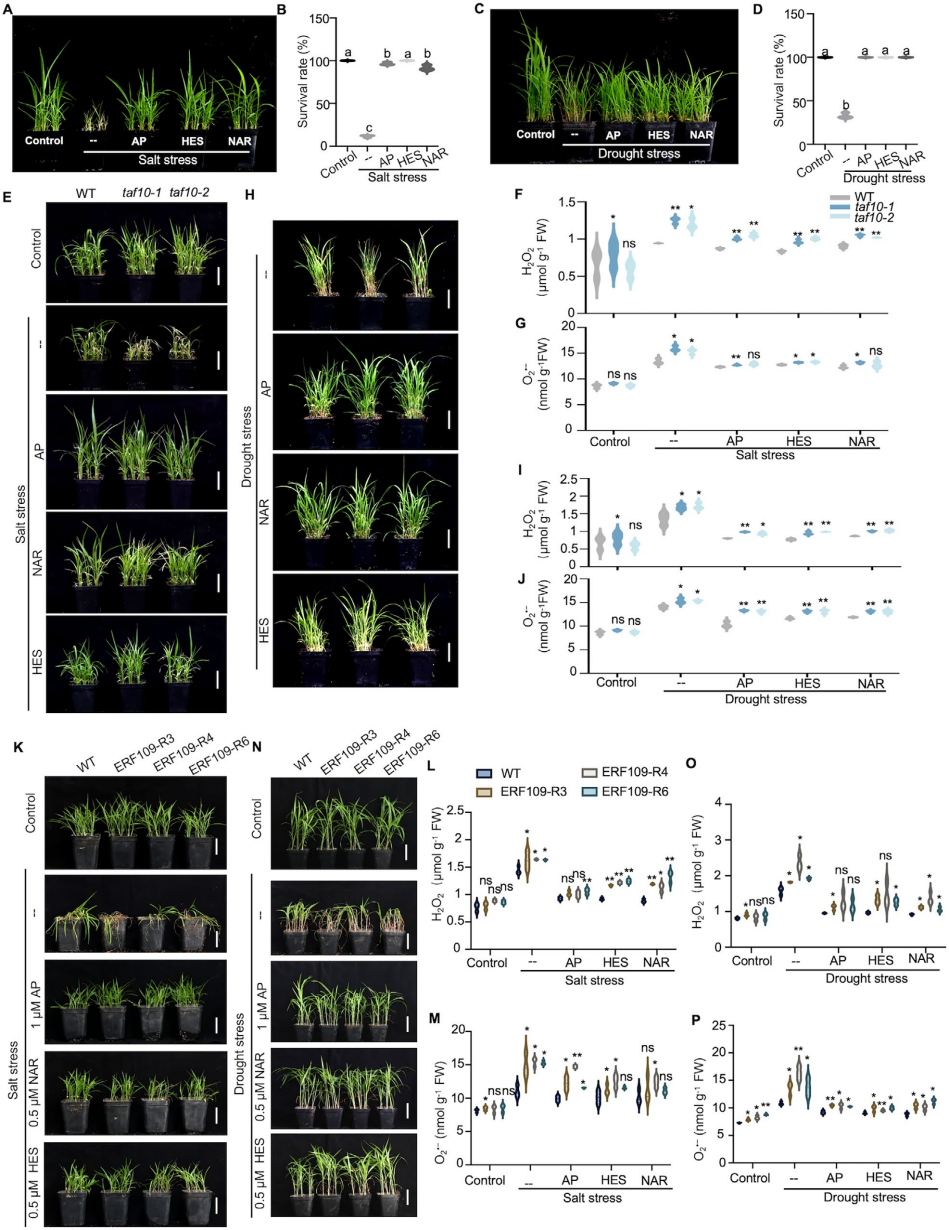
Exogenous flavonoid application enhances salt and drought resilience of foxtail millet. (A and C) Phenotypic response of WT seedlings under control, salt (300 mM NaCl) or drought stress following treatment with the flavonoid apigenin (AP), hespesretin (HES) or naringenin (NAR) at concentrations specified in Methods. (B and D) Survival rates of seedlings shown in (A and C). Data represent mean ± SD (*n* = 3 biological replicates). Different letters indicate significant differences (*p* < 0.05, Turkey's test). (E and H) Phenotypic rescue of *taf10* mutants by exogenous flavonoid application under salt (E) or drought (H) stress. Scale bars = 10 cm. (F, G, I and J) Quantitatification of H_2_O_2_ (F and I) and $O_{2}^{˙-}$ (G and J) levels in leaves of WT, *taf10* mutant and flavonoid‐treated *taf10* seedlings under salt (F and G) and drought (I and J) stress corresponding to panels (E) and (H). Data represent mean ± SD (*n* = 3). Statistical significance was determined by Student's *t*‐test (*p < 0.05, **p < 0.01). (K and N) Phenotypic comparison of WT, *ERF109*‐R and flavonoid‐treated *ERF109*‐R seedlings under salt (K) or drought (N) stress. Flavonoid (AP, HES, NAR) were applied at the indicated concentrations. (L, M, O and P) Quantitative measurements of H_2_O_2_ (L and O) and $O_{2}^{˙-}$ (M and P) levels in salt‐stressed (L and M) or drought (O and P) seedlings corresponding to treatments shown in (K) and (N). Data are presented mean ± SD (*n* = 3 biological replicates). Statistical significance was determined by Student's *t*‐test (*p < 0.05, **p < 0.01). All experiments used 21‐day‐old seedlings and were repeated with three independent biological replicates. Scale bars in (K and N) = 5 cm; others = 10 cm unless noted.

To functionally validate these observations, we treated plants with apigenin, naringenin or hesperetin, which were enriched in *SiTAF10‐*OE plants relative to the WT. We first conducted a control experiment with and without solutes and found that seedlings treated with solutes only showed no difference with water, while apigenin and naringenin promoted the growth of millet seedlings under salt (175 mM NaCl) and drought (10% PEG) stress (Figure S21). Furthermore, application of each compound of apigenin, naringenin or hesperetin conferred robust stress tolerance, improved growth and survival (Figure 6A–D and Figure S22) and diminished ROS (H_2_O_2_ and $O_{2}^{˙-}$) accumulation (Figure S23). Critically, all three compounds rescued the stress‐sensitive phenotypes of the *taf10* mutant, restoring stress tolerance close to levels in the WT and suppressing ROS accumulation (Figure 6E–J). We observed a similar rescue in *ERF109‐*R plants (Figure 6K–P), demonstrating that apigenin, naringenin and hesperetin are key functional effectors downstream of the TAF10–ERF109 module.

### 2.6 Application of Apigenin Increases the Yield of Wheat and Foxtail Millet Under Drought and Saline Soil Conditions

**FIGURE 7:**
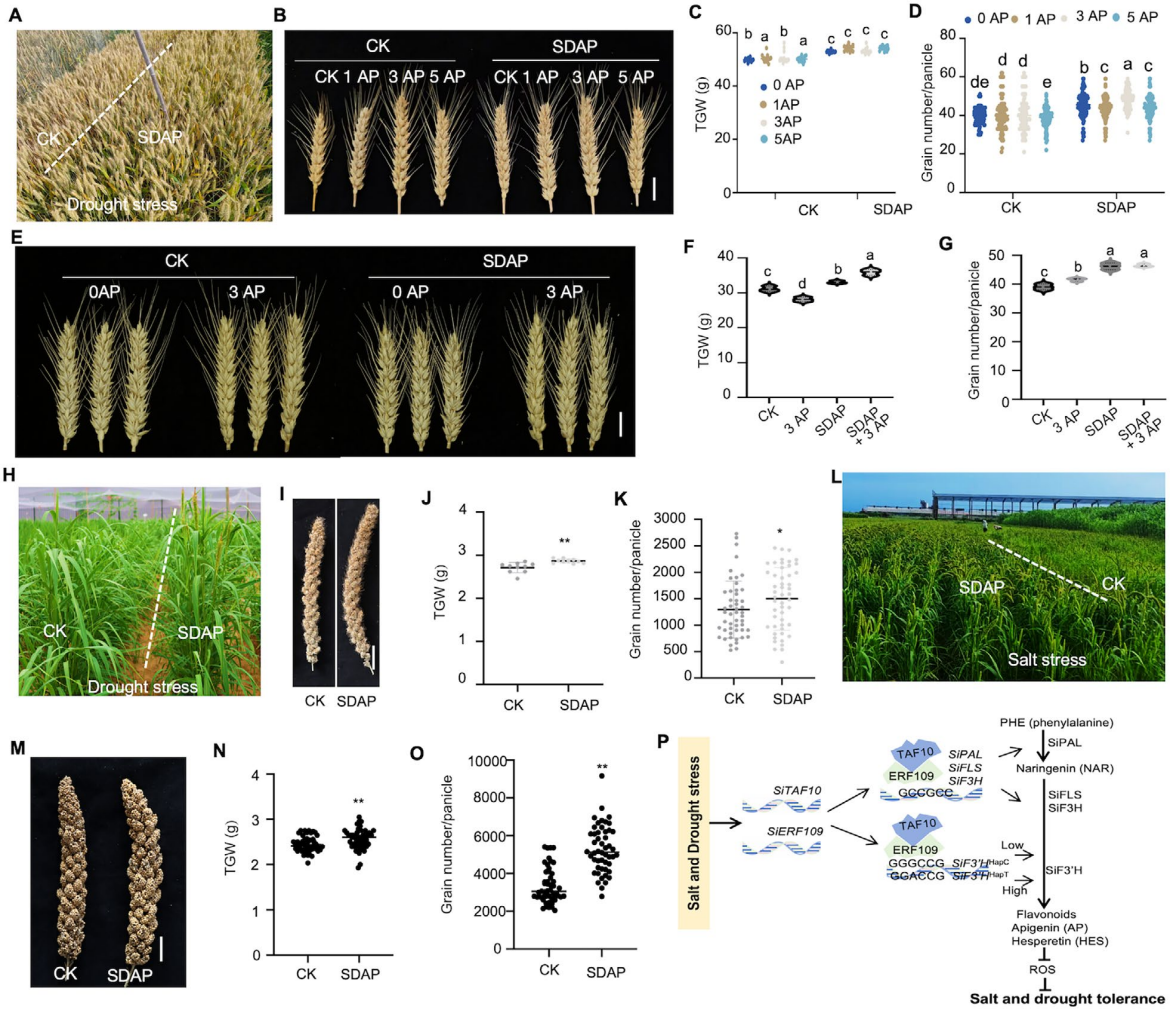
Topical application of apignin improves yield in wheat and foxtail millet under abiotic stress. (A and B) Phenotypic responses of wheat plants to the indicated concentrations of apigenin (AP) applied externally and water‐withholding at the filling stage. 1 AP, 3 AP and 5 AP denote 1 μM, 3 μM and 5 μM AP, respectively. (C and D) Thousand‐grain weight (TGW) and grain number per panicle for plants shown in (B). Different letters indicate significant differences (*p* < 0.05, Turkey's test). (E) Phenotypes of wheat seedlings grown under natural saline soil conditions in Dongying, Shandong province, China (37°24′–38°10′ N, 118°15′–119°19′ E). Seeds were treated with 1 μM AP before sowing. (F and G) TGW and grain number per panicle of wheat grown in the saline‐soil field under different AP‐treatments regimes. CK: No AP treatment throughout growth; 3 AP: Seedlings sprayed with 3 μM AP at green‐up and jointing stages (no seed treatment); SDAP: Seeds pre‐soaked with 1 μM AP (no foliar application); SDAP+3AP: Combined seed pre‐treatment with 1 μM AP and foliar spraying at green‐up and jointing stages with 3 μM AP. Different letters indicate significant differences (*p* < 0.05, Turkey's test). (H and I) Phenotypic responses of foxtail millet to external application of 1 μM AP under drought stress. (J and K) TGW and grain number per panicle for plants shown in (I). Statistical significance was determined by Student's *t*‐test (*p < 0.05, **p < 0.01). (L and M) Phenotype of foxtail millet under natural saline soil conditions in Dongying with 1 μM AP or without (CK) apigenin treatment. Scale bars = 2 cm. (N and O) TGW and grain number per panicle for plants shown in (M). Statistical significance was determined by Student's *t*‐test (*p < 0.05, **p < 0.01). (P) Proposed working model for SiERF109‐SiTAF10 module in conferring salt and drought tolerance in foxtail millet through flavonoid‐mediated ROS scavenging.

Furthermore, this finding was translated into an applied biostimulant strategy and its efficacy was evaluated in two major crops under field conditions. In wheat grown under drought stress (Tai'an), seed dressing with apigenin (SDAP), combined with apigenin spraying (AP) at the green‐up and jointing stages, significantly promoted growth and led to larger panicles, higher thousand‐grain weight (TGW) and more grains per panicle relative to untreated controls (Figure 7A–D). We identified 3 μM as the optimal concentration for treatment. An optimized protocol that combined seed dressing (1 μM) with foliar spray (3 μM) at the green‐up and jointing stages was superior to either treatment alone, increasing the yield by up to 12.3% in drought‐affected fields. Under natural saline soil (Dongying) conditions, SDAP combined with apigenin spraying at the green‐up and jointing stages significantly promoted plant height, panicle size, grain number per panicle, TGW and yield (by up to 19.6%) (Figure 7E–G).

In foxtail millet, a highly economical seed‐dressing‐alone protocol was sufficient to significantly enhance panicle size and grain yield under drought stress (Tai'an) and natural saline soil (Dongying) conditions. Treated plants exhibited larger panicle size, higher TGW and grain number per panicle compared with untreated controls and displayed a yield higher by up to 15.8% in drought‐affected fields and by up to 23.9% under natural saline soil conditions compared with untreated controls (Figure 7H–O).

The core invention of the use of apigenin to enhance crop salinity stress tolerance has been granted Chinese national patent ZL202010854231.4, confirming its innovative and significant application potential.

## 3 Discussion

Foxtail millet exhibits exceptional stress tolerance and high nutritional quality (Diao et al. 2014; Li et al. 2022), yet the genetic underpinnings of these agronomic traits remain poorly defined. Our GWAS of 731 foxtail millet natural accessions revealed a significant association between a genomic region containing *SiF3′H* and variation in salinity stress tolerance (Figure 1A). We further demonstrated that SiF3′H enhances salinity stress tolerance and broadens drought adaptability of plants. These advantages are driven by *SiF3′H*‐dependent flavonoid accumulation, which mitigates oxidative stress. This claim is firmly supported by transgenic evidence and haplotype‐specific analysis that directly link variation at the *SiF3′H* locus to distinct phenotypic and metabolic outcomes (Figure 1B–S).

Beyond identifying a key biosynthetic gene, we uncovered a previously uncharacterized transcriptional module centred on SiTAF10 and SiERF109 that acts as a master switch for stress‐inducible flavonoid production. Besides, there are several MYB binding sites, a W‐box element and other responsive elements in the *SiF3′H* promoter (Figure S3), indicating other upstream regulators for *SiF3′H* expression, which is worthy of determination in the future. While R2R3‐MYB, bHLH and WD40 proteins are recognized as canonical regulators of flavonoid and anthocyanin biosynthesis across plant species (Feller et al. 2011; Mehrtens et al. 2005; Naik et al. 2022; Stracke et al. 2007), our finding that SiTAF10 partners with SiERF109 to activate *SiF3′H* expression reveals an alternative, non‐canonical regulatory pathway dedicated to abiotic stress acclimation.

ERF‐type transcription factors constitute one of the largest transcription factor families in plants, with numerous members implicated in stress responses. For instance, the expression of 21 of the 171 *ERF*s in foxtail millet is changed by drought or salinity (Lata et al. 2014). Similar numbers have been reported in maize (Feng et al. 2020). Although a few ERFs, such as MdERF38 in apple ( *Malus domestica* ) (An et al. 2020) and SlERF.G3‐like in tomato (You et al. 2024), have been linked to flavonoid biosynthesis in these dicotyledonous species under non‐stress conditions, a role for this protein family in cereal stress responses has remained elusive. Our work establishes SiERF109 as a critical regulator that couples environmental stress signals to flavonoid‐mediated antioxidant defence in foxtail millet (Figure 2). This finding illustrates a functional divergence from the well‐characterized roles of its Arabidopsis ortholog, AtERF109, which integrates jasmonate and auxin signalling to modulate root development and regeneration (Cai et al. 2014). Indeed, JASMONATE‐ZIM‐DOMAIN PROTEIN 5 (AtJAZ5) interferes with the upregulation of *ANTHRANILATE SYNTHASE α1* (*ASA1*), a tryptophan biosynthesis gene in the auxin production pathway, by AtERF109 to promote plant regeneration (Lee et al. 2024; Zhang et al. 2019).

Furthermore, the SiTAF10–SiERF109 module delineates a previously unrecognized mechanism of transcriptional control. TAFs are generally regarded as core components of the basal transcriptional machinery, with established roles in promoter recognition, complex assembly and chromatin modulation in eukaryotes (Basehoar et al. 2004; Bhuiyan and Timmers 2019; Ruppert et al. 1993; Uffenbeck and Krebs 2006). In plants, specific TAFs have been implicated in development and stress responses, often through physical interactions with transcription factors or chromatin modifiers (Furumoto et al. 2005). Among the 15 Arabidopsis TAFs, TAF13 interacts with polycomb repressive complex 2 (PRC2) components to regulate seed development (Lindner et al. 2013), while TAF12b associates with the basic leucine zipper transcription factor bZIP60 to mediate the unfolded protein response (Kim et al. 2022). NbTAF12b of *N. benthamiana* interacts with type B response regulators (B‐RRs) to positively regulate cytokinin signalling (Guo et al. 2024). Our study demonstrates that the SiTAF10–SiERF109 module activates a specialized metabolic program under multiple stress conditions (Figures 3 and 4), illustrating a sophisticated mechanism for tailoring the general transcriptional machinery to specific environmental cues. Furthermore, phylogenetic analysis indicates that the TAF‐ERF109 module is conserved in wheat, and the promoters of *PAL* and *F3H* contain ERF‐binding elements (Figure S24), suggesting that they may play a similar role in wheat.

The SiTAF10–SiERF109 module exerts broad influence over the phenylpropanoid pathway. It upregulates the expression of 12 genes in this pathway and promotes the accumulation of diverse metabolites, such as coumarins, lignin, phenolic acids and stilbenes (Figure 4, Table S3), all of which contribute to stress acclimation (Dong and Lin 2021; Liu et al. 2020; Zhao et al. 2022). The functional relevance of this network is underscored by our observation that overexpression of *SiPAL* restores stress tolerance in the *taf10* and *ERF109*‐R backgrounds (Figure 5 and Figure S17). Highlighting the central role of the pathway modulated by SiTAF10–SiERF109 in the improvement of stress resilience in foxtail millet, even in other crops.

Flavonoids are well‐established antioxidants that alleviate ROS‐induced cellular damage under stress conditions in plant species such as rice, rapeseed and tobacco (Chen et al. 2019; Cui et al. 2014; Kim et al. 2017). Consistent with this idea, *SiF3′H*‐overexpressing lines exhibited elevated flavonoid levels and lower ROS contents under salinity or drought treatment (Figure 1J–S). Application of apigenin, naringenin or hesperetin improved the stress tolerance of wild‐type plants and rescued the hypersensitivity of lines with lower *SiERF109* or *SiTAF10* function by mitigating ROS accumulation (Figure 6 and Figures S21–S23). While apigenin and naringenin have known roles in plant stress acclimation (Ozfidan‐Konakci et al. 2020; Singh et al. 2021), most previous studies have focused on the role of hesperetin in human disease treatment (Mendes Soares et al. 2026; Ruan et al. 2026) and no effect has been found to be involved in abiotic stress in plants, our work identifies hesperetin as a previously uncharacterized effector in abiotic stress resilience.

Beyond their antioxidant activity, flavonoids such as apigenin modulate auxin transport (Chapman and Muday 2021) and serve as chemoattractants for beneficial rhizobacteria (Kumar et al. 2023; Yan et al. 2022). They stimulate and alter the physiological processes of auxin transport and distribution in roots (Buer and Muday 2004), thereby altering plant growth and development (Sharma et al. 2020). For example, flavonoids can promote PIN1 shifts in response to a gravity stimulus (Buer and Muday 2004; Peer et al. 2004; Peer and Murphy 2007; Santelia et al. 2008). It is plausible that the differential flavonoid accumulation between HapT and HapC, as well as *SiTAF10* and *SiERF109* transgenic plants could influence auxin distribution, thereby contributing to observed morphological differences. This suggests additional mechanisms that may contribute to stress tolerance and growth promotion, representing a promising area ripe for further investigation.

The most significant translational outcome of our work is the validation of apigenin as an effective, natural and field‐ready agrochemical by promoting panicle size, thousand‐grain weight (TGW) and grain number per panicle (Figure 7A–O). This achievement is particularly notable when compared to prior metabolite‐based interventions. For example, while the application of a T6P precursor spray over 4 years led to an average yield increase of 18% in wheat (Griffiths et al. 2016, 2025), and TPP spray led to grain yield increase of up to 9.8% in maize, rice or rapeseed (Luo et al. 2026). Our simple and cost‐effective protocol, combining seed coating with foliar spray, achieved substantially higher gains in wheat (up to 19.6%) under real‐field stress. Moreover, our strategy demonstrated remarkable efficacy in a second crop, foxtail millet (yield increases up to 23.9%). Highlighting its broader applicability. This metabolite‐based strategy effectively circumvents the genetic complexity of stress tolerance and avoids the regulatory and public acceptance challenges associated with transgenic crops. As a flavonoid, apigenin is biodegradable and can be produced at large scale from plant sources, making it a promising sustainable biostimulant. At a cost of only $1.76 per hectare, this highly effective and environmentally benign strategy offers a more accessible and promising route toward sustainable cultivation on drought‐affected and saline‐degraded lands, thereby contributing to global food security. Although the optimal field application concentration of apigenin has been established for wheat, further investigation is required to determine its optimal concentration for other crops. Additionally, the potential application of naringenin and hesperetin as promising sustainable biostimulants warrants further study.

In summary, we have delineated a stress‐inducible SiERF109–SiTAF10 transcriptional complex that activates flavonoid biosynthesis independently of canonical MYB‐based mechanisms, thereby enhancing ROS scavenging and stress tolerance (Figure 7P). This pathway can be leveraged through both crop genetic engineering and metabolite application. Our findings provide fundamental insights into the transcriptional regulation of stress acclimation in cereals and deliver actionable strategies for enhancing climate resilience and nutritional quality in crops.

## 4 Experimental Procedures

### 4.1 Plant Materials, Growth Conditions and Experimental Treatments

Two distinct foxtail millet ( *Setaria italica* ) cultivars were used in this study: ‘Ci846’ (chosen for high transformation efficiency) and ‘xiaomi’ (selected for its rapid growth cycle). Seeds were surface‐sterilized and sown in 8 × 8 cm pots containing a 1:1 (v/v) mixture soil and vermiculite. Plants were grown under controlled greenhouse conditions with 16‐h light/8‐h dark photoperiod, temperature 25°C, relative humidity of 60% and a light intensity of 100–150 μmol photons m^−2^ s^−1^.

Field trials were conducted using foxtail millet cultivar ‘yugu1’ and wheat ( *Triticum aestivum* ) cultivar ‘jimai22’.

### 4.2 Stress Treatments and Sample Collection

For inducible expression analysis, 7‐day‐old ‘Ci846’ seedlings were treated with 20% (w/v) polyethylene glycol 6000 (PEG6000) to stimulate drought stress, or with 200 mM NaCl in Hoagland solution to simulate salt stress. Samples were collected at 0, 3, 6, 9 and 12 h after treatment.

For tissue‐specific expression profiling, roots, stems, leaves from 14‐day‐old seedlings, panicles from 2‐month‐old plants and mature seeds were harvested. All samples were flash‐frozen in liquid nitrogen and stored at −80°C.

### 4.3 Phenotypic Analysis Under Abiotic Stress

For salt tolerance assays, 21‐day‐old seedlings were irrigated with 300 mM NaCl solution for 10 days (*n* = 10 seedlings per pot, ≥ 3 biological replicates).

For drought tolerance assessment, 21‐day‐old seedlings were subjected to 30‐day water withholding, followed by a 3‐day rewatering period to evaluate recovery capacity.

### 4.4 Physiological Measurements

Survival rates were calculated as the percentage of surviving seedlings relative to the total number of treated seedlings. Root and shoot lengths were measured using a ruler and fresh weights were determined with an electronic balance. Chlorophyll (Chl) content per gram of fresh weight was quantified according to the previously described method (Xiao et al. 2023).

ROS accumulation was assessed by histochemical staining using 3′3‐diaminobenzidine (DAB) for H_2_O_2_ and nitroblue tetrazolium (NBT) for $O_{2}^{˙-}$ and quantified with commercial kits (H_2_O_2_: BC3590; $O_{2}^{˙-}$: BC1290; Solarbio, China). Total flavonoid content was determined using a commercial assay kit (M0118A; Suzhou Mengxi Biological Medicine Technology Co. Ltd., China).

### 4.5 Exogenous Flavonoid Treatments

Twenty‐one‐day‐old seedlings were treated with apigenin (AP; 0.5 μM or 1 μM), hesperetin (HES; 0.5 μM or 5 μM) or naringenin (NAR; 0.5 μM or 2 μM). Treatments were applied concurrently with either 300 mM NaCl (salt stress) or drought stress. The same amount of solute (anhydrous ethanol) was added to the control. Each experiment was performed in three independent biological replicates.

For field applications, the results of the field experiment were obtained through two repeated experiments (2024 and 2025) at two independent locations; seeds of wheat and foxtail millet were pre‐treated with a 1 μM apigenin solution prior to sowing. Seeding rates were 300 kg/ha for wheat and 15 kg/ha for foxtail millet. Specifically, 1 L of apigenin solution was to treat either 10 kg (drought stress) or 20 kg (saline condition) of wheat seeds, and 0.5 kg (drought stress) or 1 kg (saline condition) of foxtail millet seeds. Additionally, at the turning green and jointing stages, wheat plants were foliar‐sprayed with the apigenin solution (1, 3 or 5 μM) at a rate of 150 L/ha. Tai'an (36.156° N, 117.116° E) and Dongying, China (37°24–38°10 N, 118°15–119°19 E) were selected for the drought (without watering at filling stage) and saline (0.3%–0.5% salinity) treatment, respectively.

### 4.6 GWAS

The foxtail millet accessions used for GWAS and salt tolerance analysis have been described previously (Xiao et al. 2023). GWAS were performed using mean survival rates (based on three biological replicates) and a set of 0.8 million common SNPs derived from a genome‐sequencing project (Jia et al. 2013). Analysis and model comparison were carried out with TASSEL 5.2.44 (https://tassel.bitbucket.io/). Associations with a *p*‐value ≥ 1.0 × 10^−5^ (Bonferroni‐corrected threshold) were considered statistically significant. Manhattan plots were generated using R v 3.4.4.

### 4.7 Generation of Transgenic Lines

For overexpression, full‐length coding sequences of *SiF3′H* (Seita.2G038800), *SiERF109* (Seita.6G181700) and *SiTAF10* (Seita.2G213300) were cloned into the plant expression vector pCAMBIA1305.1 via *Kpn*I/*Sal*I sites.

For CRISPR‐Cas9‐mediated knockout, sgRNAs targeting *SiTAF10* were designed using the online tool available at http://cbi.hzau.edu.cn/crispr/ and cloned into the binary vector pYLCRISPR/Cas9.

For RNA interference (RNAi), a unique 20‐bp fragment from each gene was synthesized by Shenggong Bioengineering Co. Ltd. and inserted between the *Bam*HI and *Sac*I sites of the PTCK303 vector. Compare the potential 20 bp sequences with the genomic database to eliminate those with high homology to the coding sequences of other genes and avoid off‐target effects.

All constructs were introduced into *Agrobacterium tumefaciens* strain EHA105. Foxtail millet transformation was carried out using previously established protocols (Saha and Blumwald 2016; Zhang et al. 2023). Homozygous transgenic plants were on medium containing kanamycin (50 mg L^−1^) or hygromycin (25 mg L^−1^). The *taf10* mutants were confirmed by Sanger sequencing. All primers used for vector construction were listed in Table S4.

For hairy root transformation, *SiPAL* and *SiF3′H* were cloned into the pSuper1300‐GFP vector (*Kpn*I/*Sal*I) and introduced into foxtail millet via *Agrobacterium rhizogenes* ‐mediated transformation as described (Zhang et al. 2021).

### 4.8 RNA Extraction and Quantitative RT‐PCR

Total RNA was extracted using a Universal Plant Total RNA Extraction Kit (Spin‐column‐I; BioTeke, Beijing, China) and reverse transcribed with PrimeScriptTM RT reagent kit (TaKaRa, Ohtsu, Japan) according to the manufacturer's protocols. Quantitative PCR was performed using MonAmpTM SYBRGreen qPCR Mix (Monad) on a QuantStudio6 instrument (Applied Biosystems, USA). *18S RNA* and *SiACTIN7* (Xiao et al. 2023) were used as internal controls. Each experiment included at least three biological replicates. All primers used are listed in Table S4.

### 4.9 RNA‐Sequencing

Total RNA extraction, library construction and sequencing were all carried out by Wuhan Meiwai Metabolic Technology Co. Ltd. (Wuhan, China). Subsequently, bioinformatics analysis was conducted using the online data analysis platform Meiwai Cloud (https://cloud.metware.cn/). The gene expression level was expressed in FPKM values. The screening criteria for differentially expressed genes were |fold change| ≥ 1.5 and the corrected *p*‐value < 0.05 (Tusher et al. 2001). Finally, the Unigene sequences were compared with the KEGG and GO databases using the Diamond Blastx software to complete the functional gene annotation.

### 4.10 Untargeted Metabolomics Profiling

Metabolomics analysis was performed using ultra‐performance liquid chromatography‐mass spectrometry. Thermo Fisher Van‐ quish Flex UHPLC system coupled to a mass spectrometer (Thermo Fisher Scientific, Waltham, MA; Zhang et al. 2025). Metabolite quantification was accomplished using multiple reaction monitoring (MRM) analysis by triple quadrupole mass spectrometry.

### 4.11 Statistical Analysis

Data are represented as the mean ± standard deviation (SD) of three independent replicates. Statistical significance of differences was determined using Student's *t*‐test (*p < 0.05 and **p < 0.01). Tukey's test was also used for statistical analysis and labelled with different letters (*p* < 0.05).

## Author Contributions

M.Z., C.W., C.Z. and X.D. designed the study. M.Z., K.W., J.F., Y.Z., Q.Z., H.D. and S.X. performed experiments. M.Z. and C.W. analysed the data and wrote the manuscript. G.Y., J.H., K.Y., S.Z., Q.H. and G.J. provided experimental suggestions and revised the manuscript.

## Funding

This work was supported by the National Key Research and Development Program of China, 2022YFD1201704. The Natural Science Foundation of China, 32472064, 32241039, 32401886.

## Conflicts of Interest

The authors declare no conflicts of interest.

## Supporting information


**Figure S1:** Molecular characterization of *SiF3′H* transgenic foxtail millet lines.
**Figure S2:** Stress‐responsive and tissue‐specific expression of *SiF3′H*.
**Figure S3:** Analysis of cis‐acting regulatory elements in the *SiF3′H* promoter.
**Figure S4:** Phylogenetic analysis and stress‐responsive expression of *SiERFs*.
**Figure S5:** Stress‐responsive and tissue‐specific expression of *SiERF109*.
**Figure S6:** Identification of ERF109 transgenic foxtail millet lines.
**Figure S7:** Regulation of *SiF3′H* expression by SiERF109.
**Figure S8:** BiFC analysis of the SiTAF10‐SiERF109 interaction.
**Figure S9:** Expression analysis of *SiTAF10* in WT, transgenic and CRISPR/Cas9‐induced mutant lines.
**Figure S10:** *SiTAF10* enhances antioxidant capacity in foxtail millet seedlings under abiotic stress.
**Figure S11:** Total flavonoid content in WT, SiTAF10‐OE and *taf10* mutant lines under control conditions.
**Figure S12:** Transcriptome profiling reveals *SiTAF10*‐mediated gene regulatory networks.
**Figure S13:** Identification of SiTAF10 binding sites in promoters of flavonoid biosynthesis genes.
**Figure S14:** Stress‐responsive and tissue‐specific expression of *SiPAL*, *SiFLS* and *SiF3H*.
**Figure S15:** TAF10 does not directly activate flavonoid pathway gene expression.
**Figure S16:** Promoter architecture and transcriptional regulation of flavonoid biosynthesis genes by SiERF109.
**Figure S17:** *SiPAL* rescues salt and drought sensitivity of *SiERF109*‐RNAi and *taf10* mutant seedlings through flavonoids‐mediated ROS scavenging.
**Figure S18:** Identification and analysis of differentially accumulated metabolites between *SiTAF10*‐OE3 and WT lines.
**Figure S19:** *SiTAF10* promotes flavonoid accumulation in foxtail millet leaves.
**Figure S20:** *SiTAF10*, *SiERF109*, *SiF3′H* positively regulate flavonoid accumulation in foxtail millet seeds.
**Figure S21:** Effects of solute (anhydrous ethanol) on the growth of foxtail millet seedlings.
**Figure S22:** Effects of flavonoids on abiotic stress tolerance in foxtail millet seedlings.
**Figure S23:** ROS detection in flavonoid‐treated foxtail millet plants.
**Figure S24:** Evolutionary tree analysis of TAF10 and ERF109 and analysis of cis‐acting regulatory elements in the wheat *TaPALs* and *TaF3H* promoters.

**Table S1:** SNP loci associated with salt‐tolerance in foxtail millet.

**Table S2:** Potential proteins interacting with SiERF109.

**Table S3:** Metabolites detected in *SiTAF10*‐OE3 and WT leaves under normal, drought and salt stress conditions.

**Table S4:** Primers used in this study.

## Data Availability

All data supporting the findings of this study are included in the manuscript and its Supporting Information. Raw sequencing data have been deposited in the NCBI SRA (Sequence Read Archive) database under accession number PRJNA1212689.
