# Supplementary material for: A TAF10‐ERF109 Transcriptional Module Directs Flavonoid‐Based Stress Resilience and Yield Enhancement in Foxtail Millet and Wheat
Source: Plant Biotechnol J. 2026 Jun 26:10.1111/pbi.70711. Online ahead of print. doi: 10.1111/pbi.70711 (PMC13398824; doi:10.1111/pbi.70711)
Supplement: Supplementary file 1 — Figure S1: Molecular characterization of SiF3′H transgenic foxtail millet lines. Figure S2: Stress‐responsive and tissue‐specific expression of SiF3′H. Figure S3: Analysis of cis‐acting regulatory elements in the SiF3′H promoter. Figure S4: Phylogenetic analysis and stress‐responsive expression of SiERFs. Figure S5: Stress‐responsive and tissue‐specific expression of SiERF109. Figure S6: Identification of ERF109 transgenic foxtail millet lines. Figure S7: Regulation of SiF3′H expression by SiERF109. Figure S8: BiFC analysis of the SiTAF10‐SiERF109 interaction. Figure S9: Expression analysis of SiTAF10 in WT, transgenic and CRISPR/Cas9‐induced mutant lines. Figure S10: SiTAF10 enhances antioxidant capacity in foxtail millet seedlings under abiotic stress. Figure S11: Total flavonoid content in WT, SiTAF10‐OE and taf10 mutant lines under control conditions. Figure S12: Transcriptome profiling reveals SiTAF10‐mediated gene regulatory networks. Figure S13: Identification of SiTAF10 binding sites in promoters of flavonoid biosynthesis genes. Figure S14: Stress‐responsive and tissue‐specific expression of SiPAL, SiFLS and SiF3H. Figure S15: TAF10 does not directly activate flavonoid pathway gene expression. Figure S16: Promoter architecture and transcriptional regulation of flavonoid biosynthesis genes by SiERF109. Figure S17: SiPAL rescues salt and drought sensitivity of SiERF109‐RNAi and taf10 mutant seedlings through flavonoids‐mediated ROS scavenging. Figure S18: Identification and analysis of differentially accumulated metabolites between SiTAF10‐OE3 and WT lines. Figure S19: SiTAF10 promotes flavonoid accumulation in foxtail millet leaves. Figure S20: SiTAF10, SiERF109, SiF3′H positively regulate flavonoid accumulation in foxtail millet seeds. Figure S21: Effects of solute (anhydrous ethanol) on the growth of foxtail millet seedlings. Figure S22: Effects of flavonoids on abiotic stress tolerance in foxtail millet seedlings. Figure S23: ROS detection in flavonoid‐tre [file PBI-9999-0-s001.docx]

**
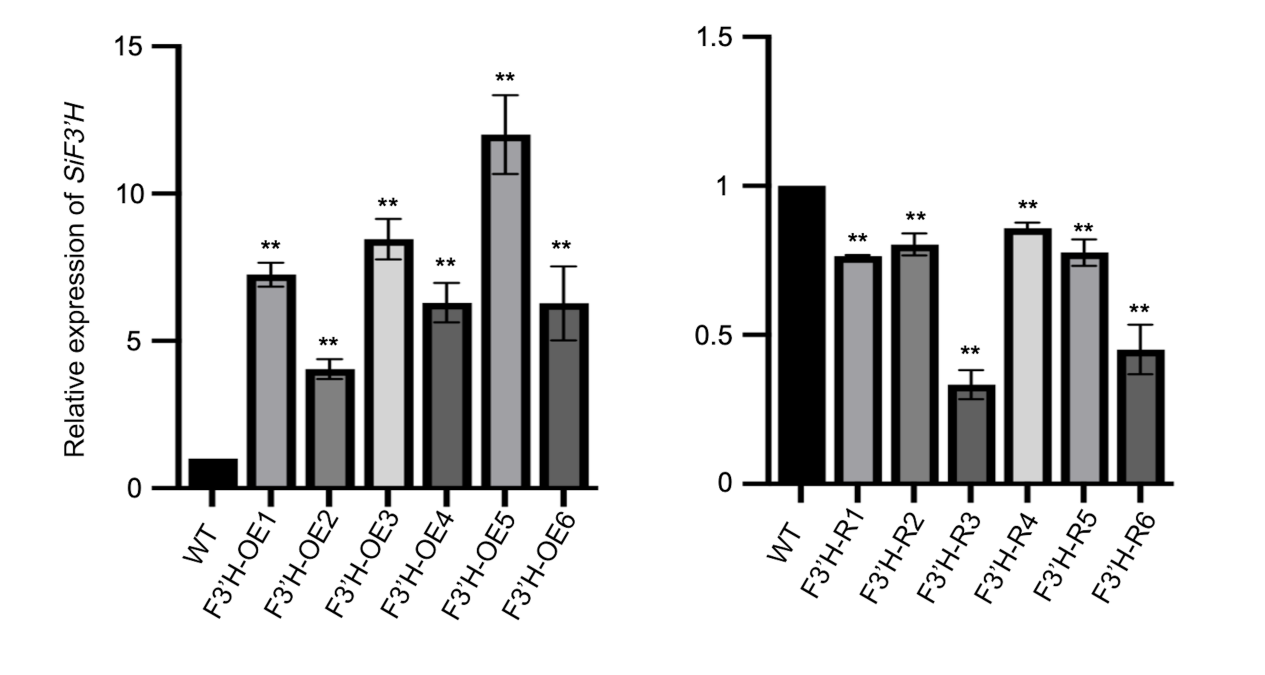
**

**Supplementary Figure 1. Molecular characterization of *SiF3'H* transgenic foxtail millet lines.** Data are mean ± SEM from three biological replicates. Statistical significance is denoted as *P < 0.05 and **P < 0.01 (Student's *t*-test).


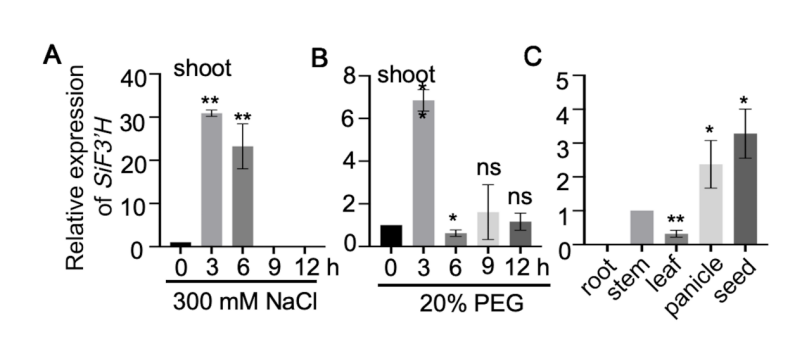


**Supplementary Figure 2. Stress-responsive and tissue-specific expression of *SiF3'H*.**

**(A)** *SiF3'H* expression in shoot under control, salt stress (300 mM NaCl for 0-24 h), as determined by RT-qPCR. **(B)** RT-qPCR analysis of *SiF3'H* expression in shoots under osmotic stress (stimulated by 20% PEG). Data are represented as mean ± SD (n = 3 biological replicates). *P < 0.05, **P < 0.01 (Student's *t*-test vs. respective controls). **(C)** Tissue-specific expression profile of *SiF3'H* in panicle, leaves, stem, roots, and developing seeds. Data represent mean ± SD (n = 3).


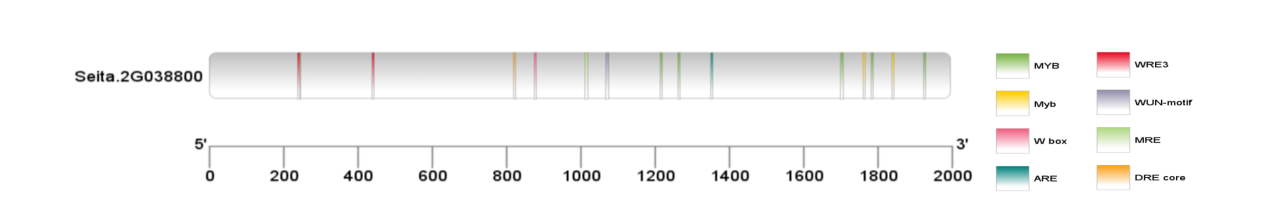


**Supplementary Figure 3. Analysis of cis-acting regulatory elements in the *SiF3'H* promoter.** Schematic representation of the 2,000 bp promoter region of *SiF3'H*, highlighting predicted cis-regulatory elements. Key elements identified include: MYB binding sites (MYB), W-box element (W box), Anaerobic response elements (ARE), Wound-responsive elements (WRE3), Wound-inducible motifs (WUN-motif), Light-responsive elements (MRE), and Dehydration-responsive elements (DRE core).


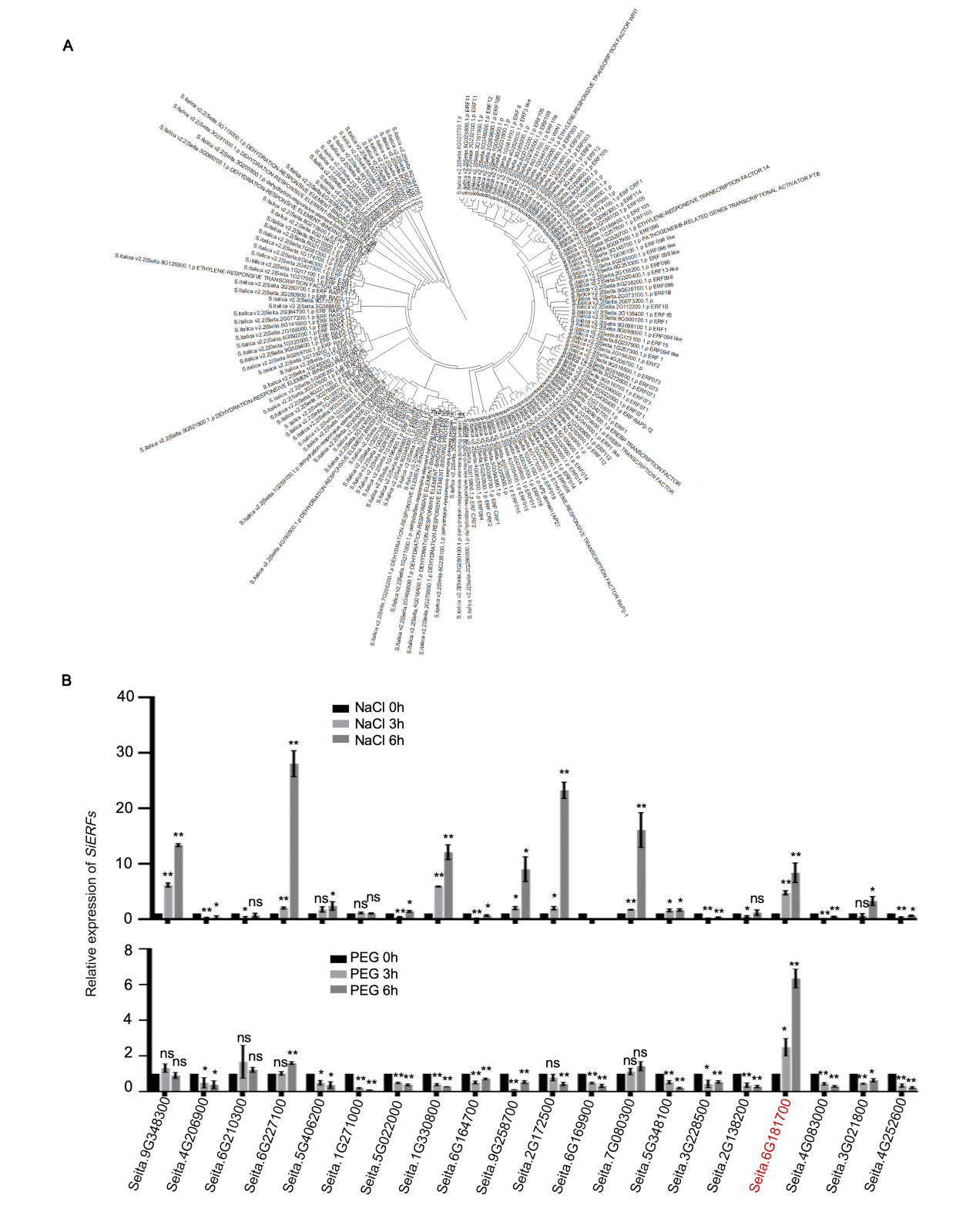


**Supplementary Figure 4. Phylogenetic analysis and stress-responsive expression of *SiERFs*. (A)** Evolutionary tree analysis of ERF transcription factors in foxtail millet. **(B)** Temporal expression profiles of *SiERF109* in shoot tissues under stress conditions. Expression dynamics were assessed by RT-qPCR under salt stress (300 mM NaCl) and osmotic stress (stimulated by20% PEG) at 0, 3 and 6 h post-treatment. Data represent mean ± SD (n = 3 biological replicates). *P < 0.05, **P < 0.01 versus 0 h control (Student's *t-*test).


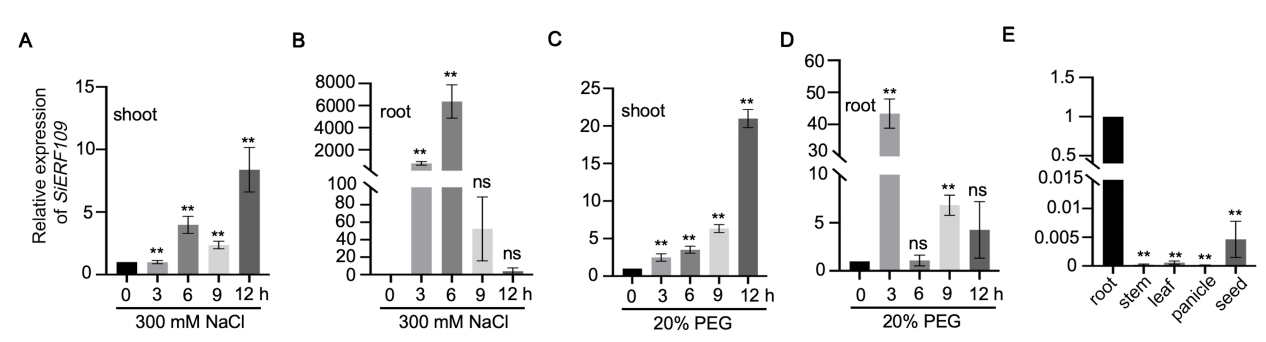


**Supplementary Figure 5. Stress-responsive and tissue-specific expression of *SiERF109*.**

**(A-D)** Temporal expression dynamics of *SiERF109* under salt and drought stresses, as determined by qRT‑PCR. Expression levels were analyzed separately in shoot and root tissues: **(A)** shoots under salt stress (300 mM NaCl); **(B)** roots under salt stress; **(C)** shoots under drought stress (10-day water withholding); and **(D)** roots under drought stress. Time-course data are shown at 0, 6, 12, and 24 h post-stress. Data represent mean ± SD (n = 3 biological replicates). *P < 0.05, **P < 0.01 versus the 0 h control (Student’s *t*-test). **(E)** Tissue-specific expression profile of *SiERF109* in panicle, leaves, stem, roots, and developing seeds. Data represent mean ± SD (n = 3).


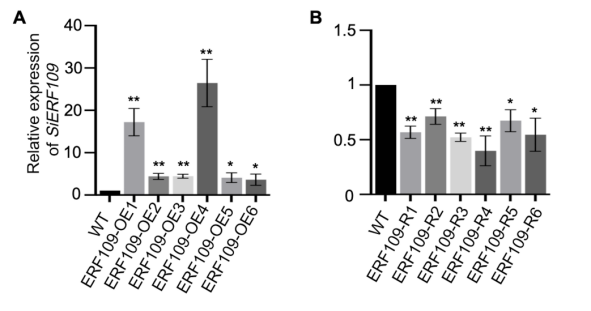


**Supplementary Figure 6.** **Identification of *ERF109* transgenic foxtail millet lines.**

**(A** and **B)** Molecular characterization of *SiERF109* transgenic foxtail millet lines. Data represent mean ± SEM from three biological replicates. Statistical significance is indicated as *P < 0.05 and **P < 0.01 (Student's *t*-test).

**
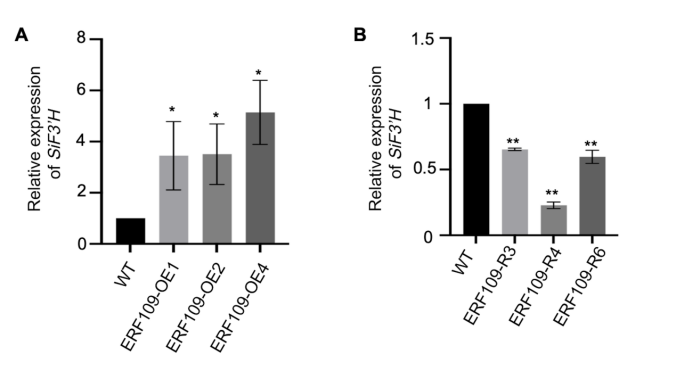
**

**Supplementary Figure 7. Regulation of *SiF3'H* expression by SiERF109.**

Data represent mean ± SEM (n = 3 biological replicates). Statistical significance versus the WT control is indicated as *P < 0.05 and **P < 0.01 (Student's *t*-test).


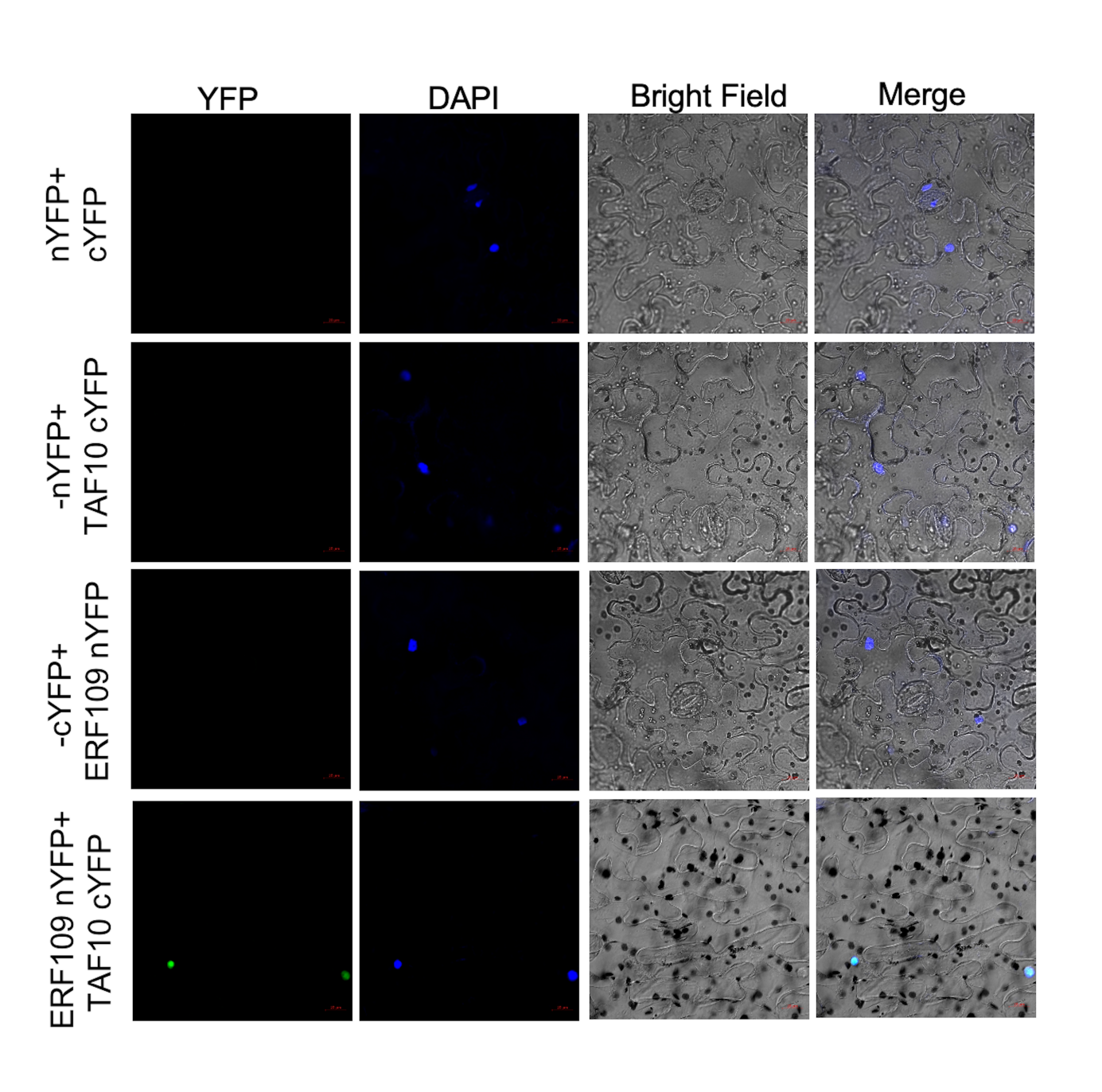


**Supplementary Figure 8.** **BiFC analysis of the SiTAF10-SiERF109 interaction.**

Bimolecular fluorescence complementation (BiFC) assays demonstrating the *in vivo* interaction between SiTAF10 and SiERF109, which localizes to the nucleus. No fluorescent signal was observed in the negative control (cYFP + SiERF109-nYFP). The YFP signal from the interaction co-localized with nuclear markers, as shown in bright field and DAPI.


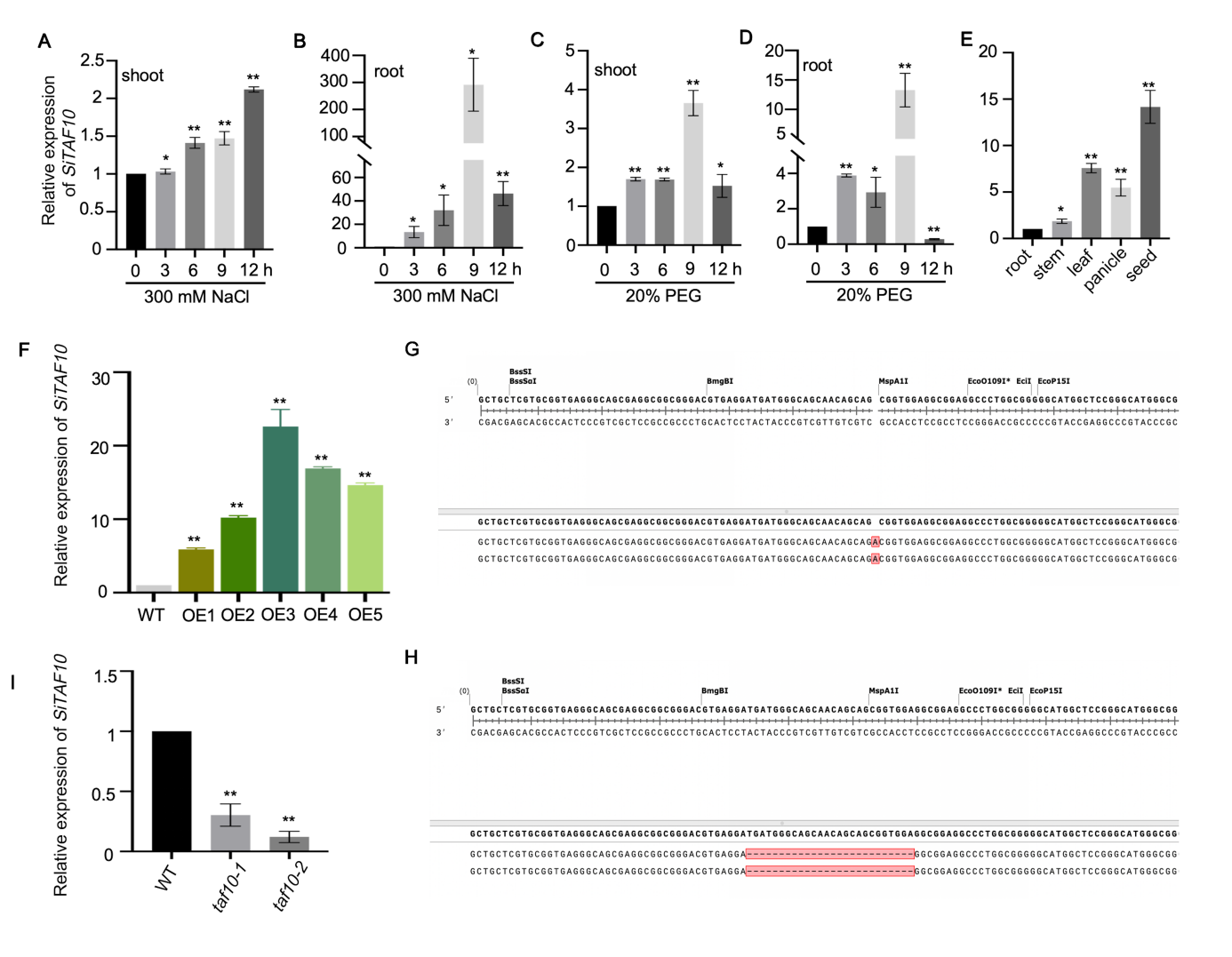


**Supplementary Figure 9.** **Expression analysis of *SiTAF10* in WT, transgenic and CRISPR/Cas9-induced mutant lines.**

**(A-D)** Time-course RT-qPCR analysis of *SiTAF10* expression in shoots and roots under salt (300 mM NaCl) and drought (20% PEG) stress at 0, 6, 12, and 24 h post-treatment. Data represent mean ± SD (n = 3 biological replicates). *P < 0.05, **P < 0.01 versus respective 0 h controls (Student's *t*-test). **(E)** Tissue-specific expression profile of *SiTAF10* in panicle, leaves, stem, roots, and developing seeds. Data represent mean ± SD (n = 3). **(F)** Validation of *SiTAF10* overexpression (OE) lines by RT-qPCR. Data represent mean ± SD (n = 3 biological replicates). **P < 0.01 versus WT (Student's *t*-test). **(G** and **H)** Sequencing chromatograms of CRISPR/Cas9-induced *taf10* mutants. Red boxes highlight the edited sites. **(I)** Validation of *taf10-1* and *taf10-2* mutants by RT-qPCR. Data represent mean ± SD (n = 3 biological replicates). **P < 0.01 versus WT (Student's *t*-test).


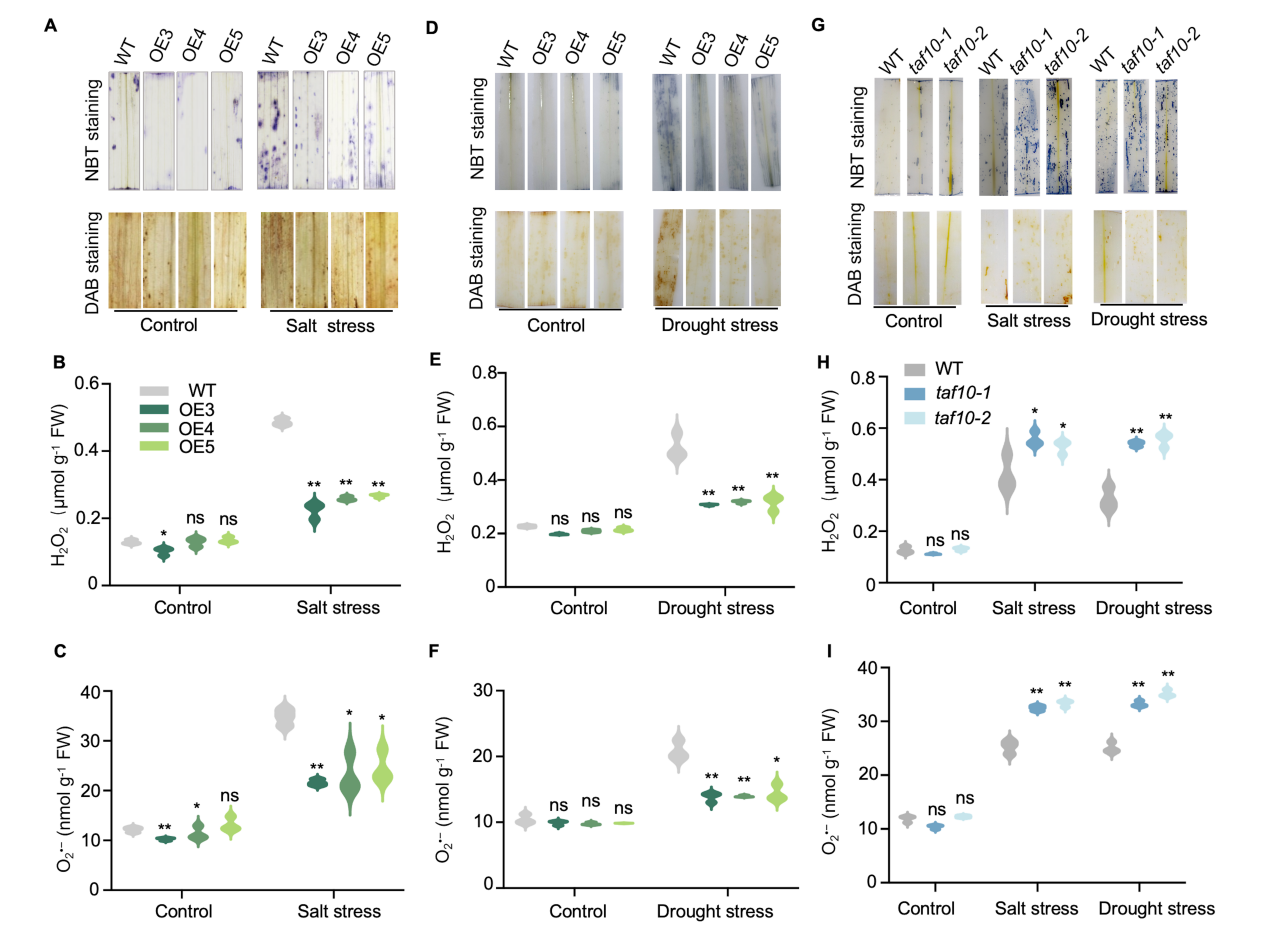


**Supplementary Figure 10. *SiTAF10* enhances antioxidant capacity in foxtail millet seedlings under abiotic stress.**

**(A)** Histochemical detection of reactive oxygen species (ROS) in *SiTAF10*-OE and wild-type leaves under control conditions and after 48 h of 300 mM NaCl treatment. ROS were visualized by NBT staining for O_2_^•−^ and DAB staining for H_2_O_2_. **(B** and **C)** Quantitative measurement of H_2_O_2_ content **(B)** and O_2_^•−^production **(C)** in leaves from **(A)**. Data represent mean ± SD (n = 3). **P < 0.01 (Student's *t*-test). **(D)** ROS staining in *SiTAF10*-OE and WT under well-watered conditions and after 10-day drought stress. **(E** and **F)** Quantitative measurement of H_2_O_2_ content **(E)** and O_2_^•−^ production **(F)** in leaves from **(D)**. Data represent mean ± SD (n = 3). **(G)** Comparative ROS accumulation in *taf10* mutants and wild-type under normal growth, NaCl stress and drought stress. **(H** and **I)** Spectrophotometric quantification of H_2_O_2_ **(H)** and O_2_^•−^ **(I)** levels in plants form **(G)**. Data represent mean ± SD (n = 3). **P < 0.01 (Student's *t*-test).

**
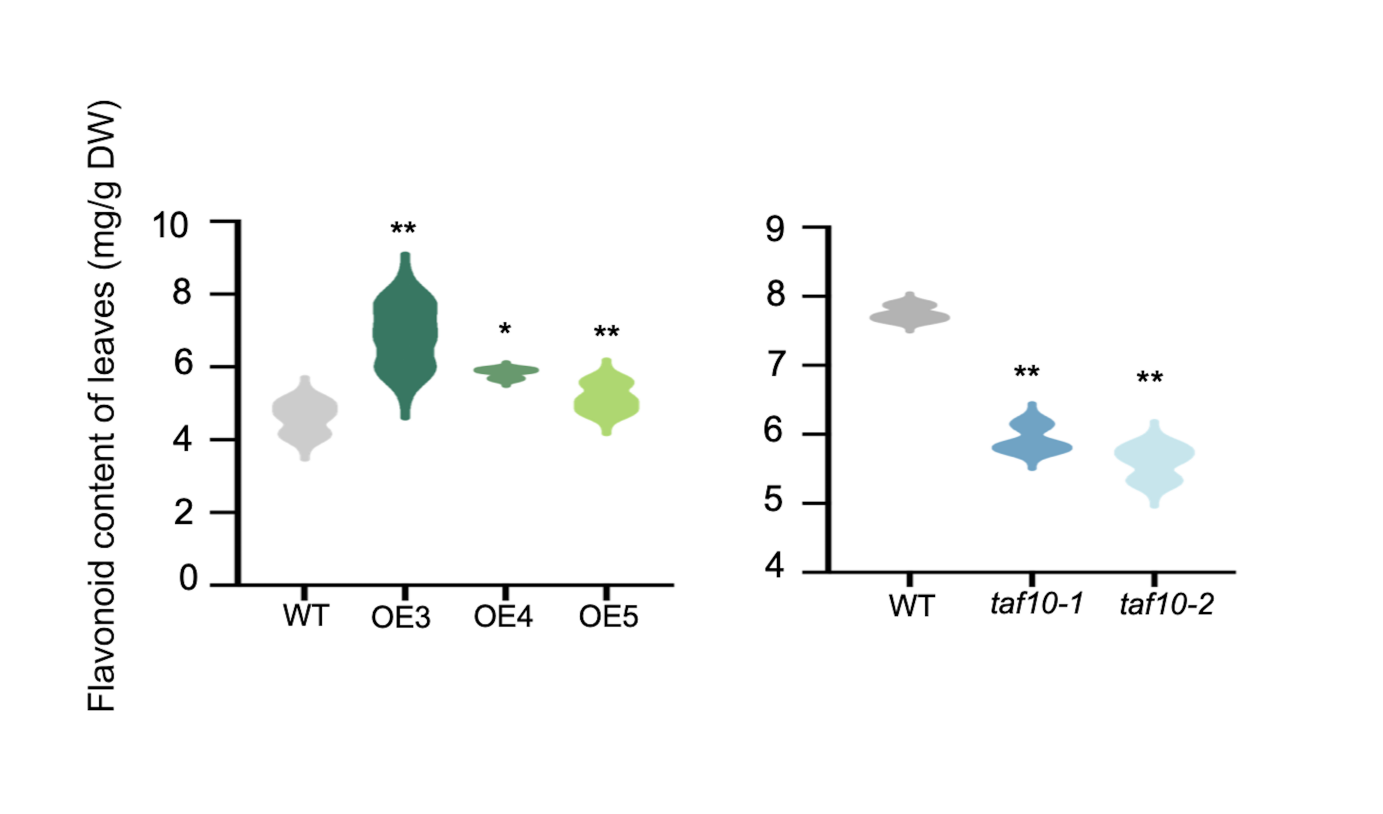
Supplementary Figure 11. Total flavonoid content in WT, *SiTAF10*-OE, and *taf10* mutant lines under control conditions.** Data represent mean ± SD (n = 3 biological replicates). **P < 0.01 versus WT (Student's *t*-test).


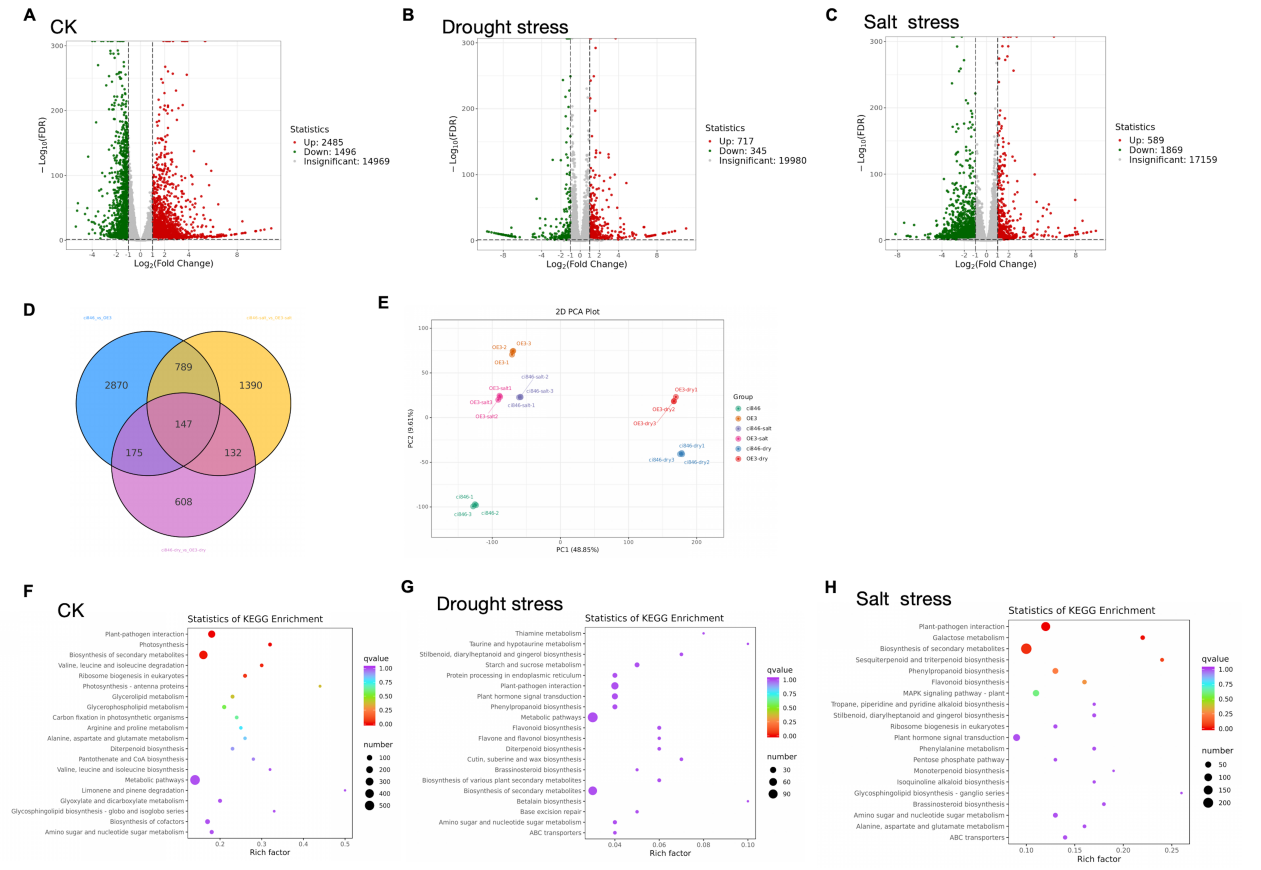


**Supplementary Figure 12. Transcriptome profiling reveals *SiTAF10*-mediated gene regulatory networks.**

**(A-C)** Volcano plots of differentially expressed genes (DEGs) in *SiTAF10*-OE versus 'Ci846' (WT) under **(A)** normal, **(B)** drought (10-day water withholding)**,** and **(C)** salt (300 mM NaCl, 24 h) conditions. Green and red dots represent down- and upregulated genes, respectively. Dashed lines indicate significance thresholds. **(D)** Venn diagram showing unique and shared DEGs across the three conditions. **(E)** Principal Component Analysis (PCA) of transcriptomes from the indicated groups. Ellipses represent 95% confidence intervals (n=3 biological replicates). **(F-H)** KEGG pathway enrichment analysis of DEGs (top 20 enriched pathways) under **(F)** normal, **(G)** drought, and **(H)** salt conditions. Bar colors indicate -log_10_ (P-value); pathway names shown on the right.


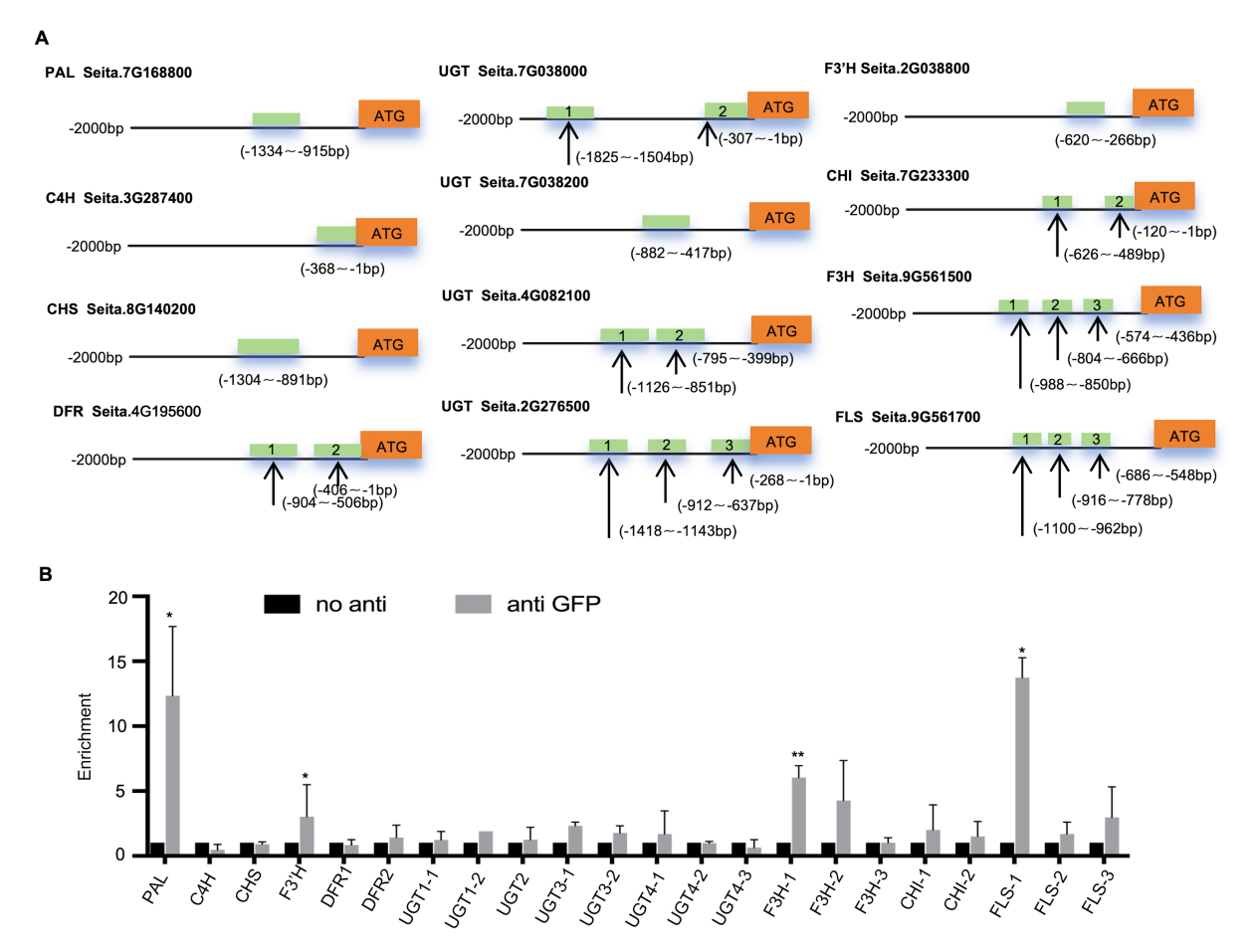


**Supplementary Figure 13. Identification of SiTAF10 binding sites in promoters of flavonoid biosynthesis genes.**

**(A)** Schematic illustration of potential regions within the promoters of flavonoid biosynthetic genes. **(B)** ChIP-qPCR validation of SiTAF10 binding to promoter regions of flavonoid biosynthetic genes. Data represent mean ± SD (n = 3 biological replicates). *P < 0.05, **P < 0.01 versus IgG control (Student's *t*-test).


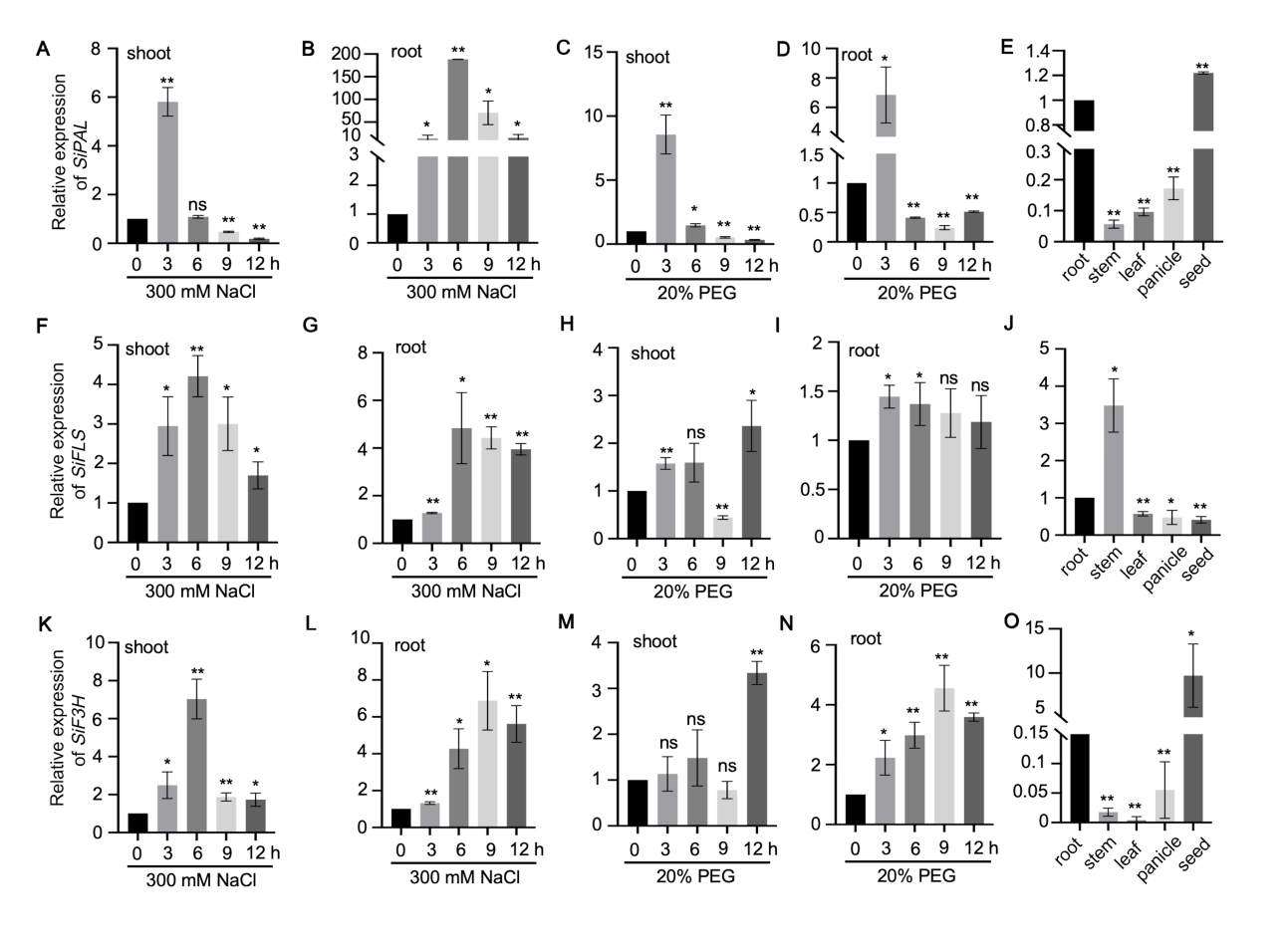


**Supplementary Figure 14.** **Stress-responsive and tissue-specific expression of *SiPAL*, *SiFLS* and *SiF3H*.**

**(A-D, F-I, K-N)** Time-course RT-qPCR analysis of *SiPAL*, *SiFLS*, and *SiF3H* expression in shoots and roots under salt (300 mM NaCl) and drought (20% PEG) stress at 0, 6, 12, and 24 h post-treatment. Data represent mean ± SD (n = 3 biological replicates). *P < 0.05, **P < 0.01 versus respective 0 h controls (Student's *t*-test). **(E, J, O)** Tissue-specific expression patterns of of *SiPAL*, *SiFLS*, and *SiF3H* in panicle, leaves, stem, roots, and developing seeds. Data represent mean ± SD (n = 3).


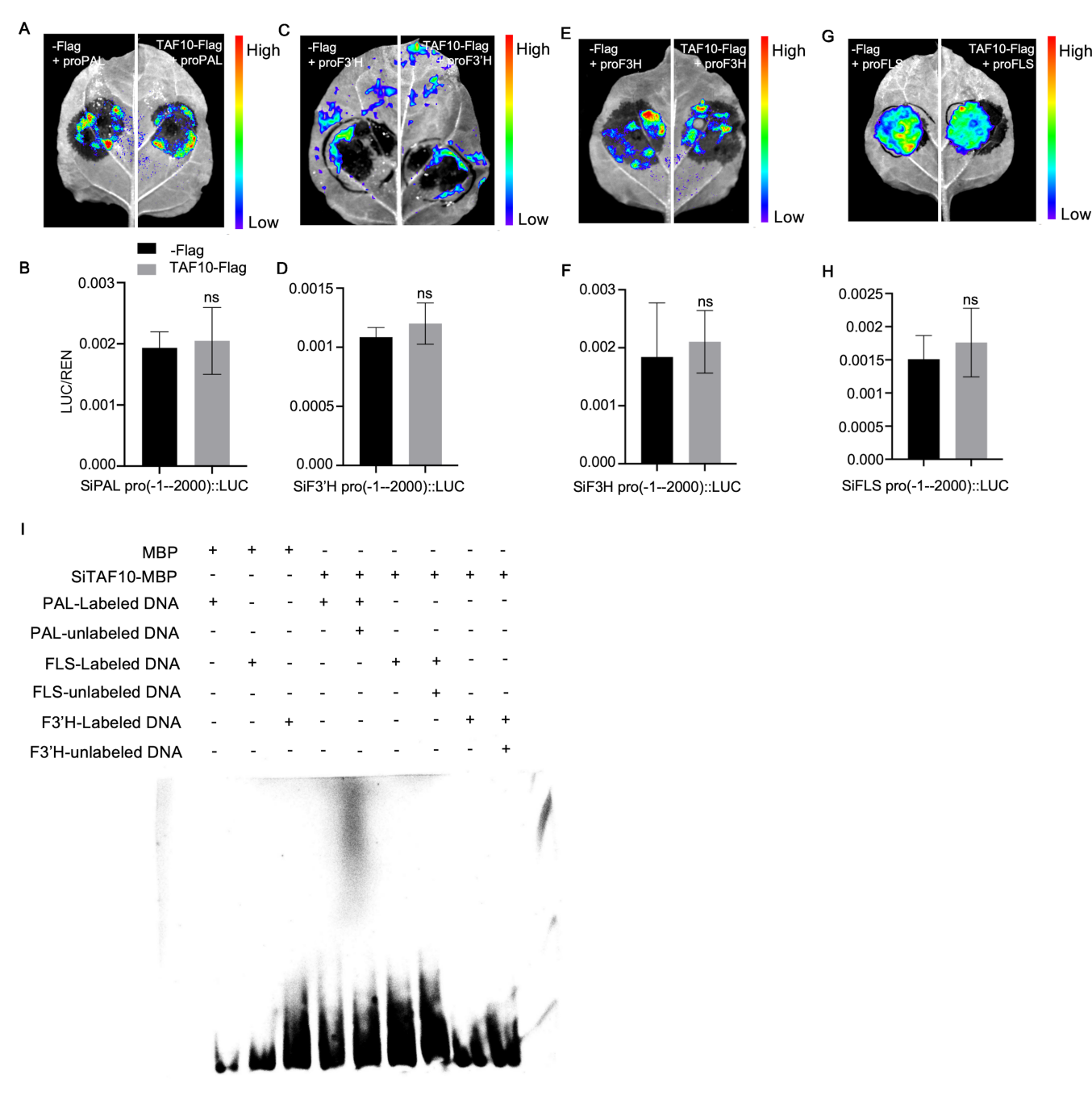


**Supplementary Figure 15.** **TAF10 does not directly activate flavonoid pathway gene expression.**

**(A, C, E, G)** Dual-luciferase reporter assay in *N. benthamiana* leaves co-infiltrated with *Agrobacterium* harboring SiTAF10-FLAG or an empty FLAG vector together with promoter-LUC reporters of *SiPAL*, *SiF3'H*, *SiF3H*, and *SiFLS* (each containing ~ 2000 bp upstream sequences). Luminescence images were taken 48 h after infiltration following luciferin application. **(B, D, F, H)** Quantification of dual-luciferase activity showing no significant activation of *SiPAL*，*SiF3'H*，*SiF3H* and *SiFLS* promoters by SiTAF10. Activities normalized to the internal 35S:REN control (LUC/REN ratio). Data represent mean ± SD (n = 3 biological replicates). ns, not significant (Student's *t*-test). **(I)** EMSA analysis of SiTAF10 binding to biotin-labeled probe and unlabeled competitors. The results demonstrate no direct binding of SiTAF10 to core promoter elements of the tested genes.


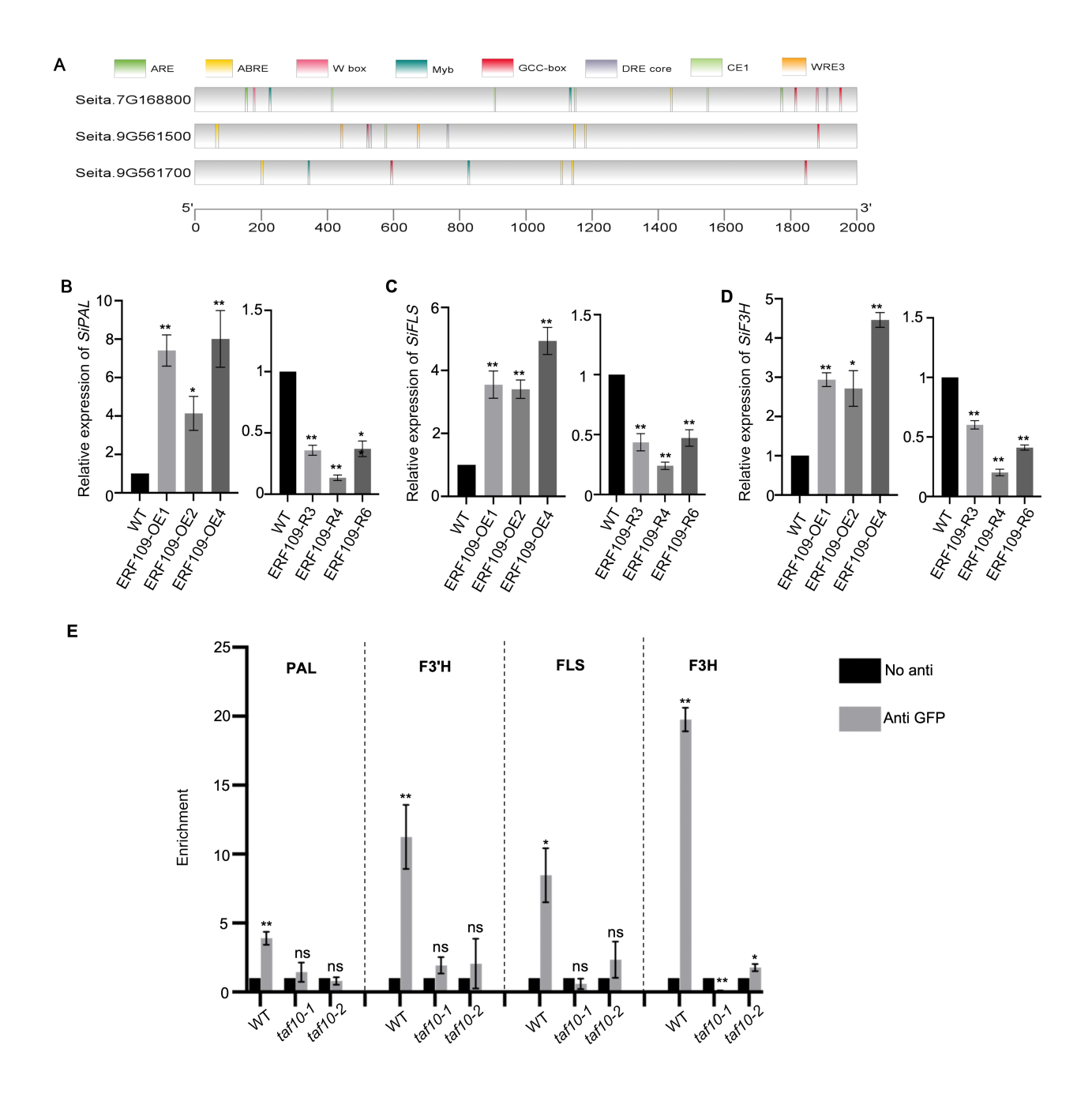


**Supplementary Figure 16.** **Promoter architecture and transcriptional regulation of flavonoid biosynthesis genes by SiERF109.**

**(A)** Schematic representation of cis-regulatory elements within the ~ 2000 bp promoter regions of *SiPAL*, *SiF3H*, and *SiFLS*. Key elements include: anaerobic response elements (ARE), W-box elements (W box), ABA-responsive elements (ABRE), MYB binding sites (Myb), GCC-boxes, Dehydration-responsive elements (DRE core), Coupling element 1 (CE1), and Wound-responsive elements (WRE3). **(B-D)** RT-qPCR analysis of flavonoid biosynthetic gene expressions in *SiERF109* transgenic lines: *SiPAL* **(B)**, *SiF3H* **(C)**, and *SiFLS* **(D)**. **(E)** ChIP-qPCR validation of SiERF109 binding to promoter regions of flavonoid biosynthetic genes in WT, *taf10-1*, and *taf10-2* hairy roots. Data represent mean ± SD (n = 3 biological replicates). *P < 0.05, **P < 0.01 vs. WT (Student's *t*-test).


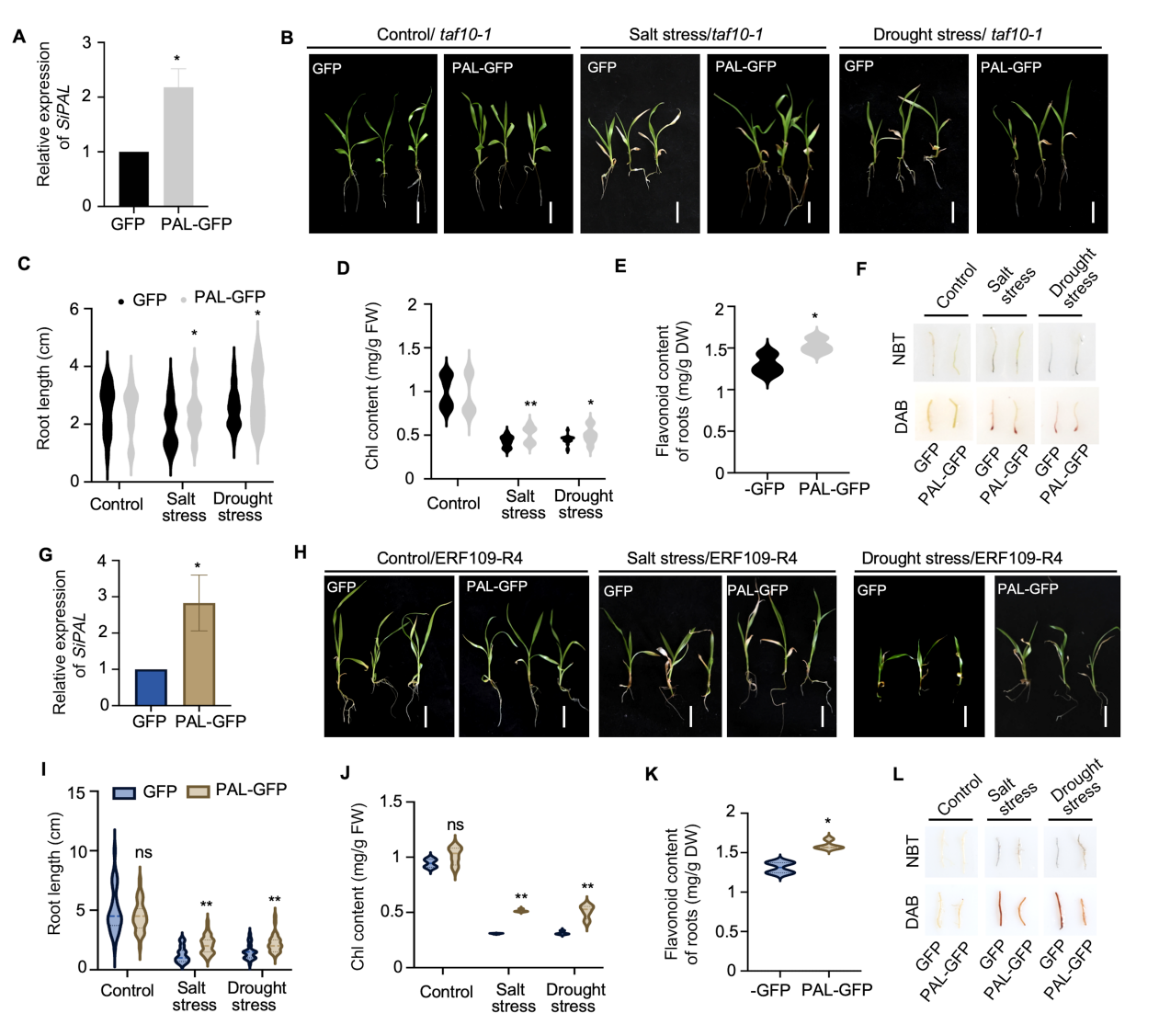


**Supplementary Figure 17. *SiPAL* rescues salt and drought sensitivity of *SiERF109*-RNAi and *taf10* mutant seedlings through flavonoids-mediated ROS scavenging.**

(**A** and **G)** *SiPAL* transcript levels in control (GFP) and *SiPAL*-overexpressing (PAL-GFP) hairy roots. Data represent mean ± SD (n = 3). **(B** and **H)** Phenotypic rescue of *SiERF109*-RNAi **(B)** and *taf10* mutant **(H)** seedlings by *SiPAL*-GFP hairy roots under control conditions, salt stress (300 mM NaCl), and drought stress. Scale bars = 2 cm. **(C, D, I, J)** Quantitative analysis of stress responses of seedlings shown in **(B** and **H),** based on primary root length **(C** and **I),** and chlorophyll (Chl) content **(D** and **J)**. **(E** and **K)** Total flavonoid accumulation in hairy roots of control and PAL-GFP transgenic *SiERF109*-R **(E)** and *taf10* mutant **(K)**. **(F** and **L)** ROS detection in hairy roots by NBT staining (O_2_^•−^) and DAB staining (H_2_O_2_) under control and stress conditions. Statistical significance: *P < 0.05, **P < 0.01; ns, not significant (Student's *t*-test). All experiments included three biological replicates.


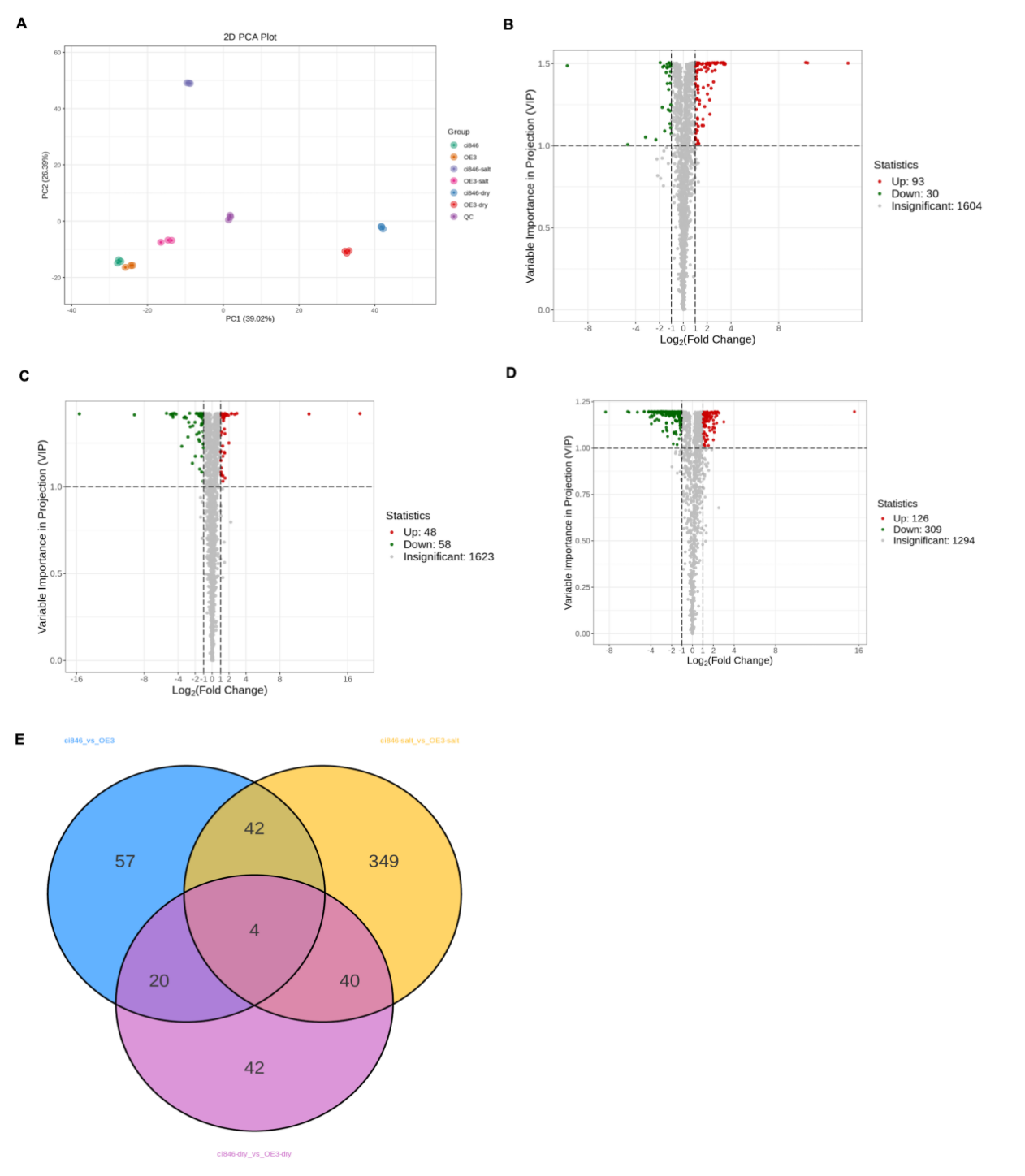


**Supplementary Figure 18. Identification and analysis of differentially accumulated metabolites between *SiTAF10*-OE and WT lines**.

**(A)** Principal component analysis (PCA) of metabolites across experimental groups. ‘Ci846’ (WT) and ‘OE3’ represent untreated seedlings; ‘Ci846-salt’ and ‘OE3-salt’ denote seedlings under salt stress; ‘Ci846-dry’ and ‘OE3-dry’ indicate seedlings under drought stress. (**B-D)** Volcano plots of differentially accumulated metabolites under **(B)** normal, **(C)** drought, and **(D)** salt stress conditions. Green and red dots represent down- and up-regulated metabolites, respectively (threshold: |log2FC| > 1, P < 0.05). **(E)** Venn diagram illustrating overlapping and unique differentially accumulated metabolites in WT and *SiTAF10*-OE lines under normal, drought, and salt stress conditions.


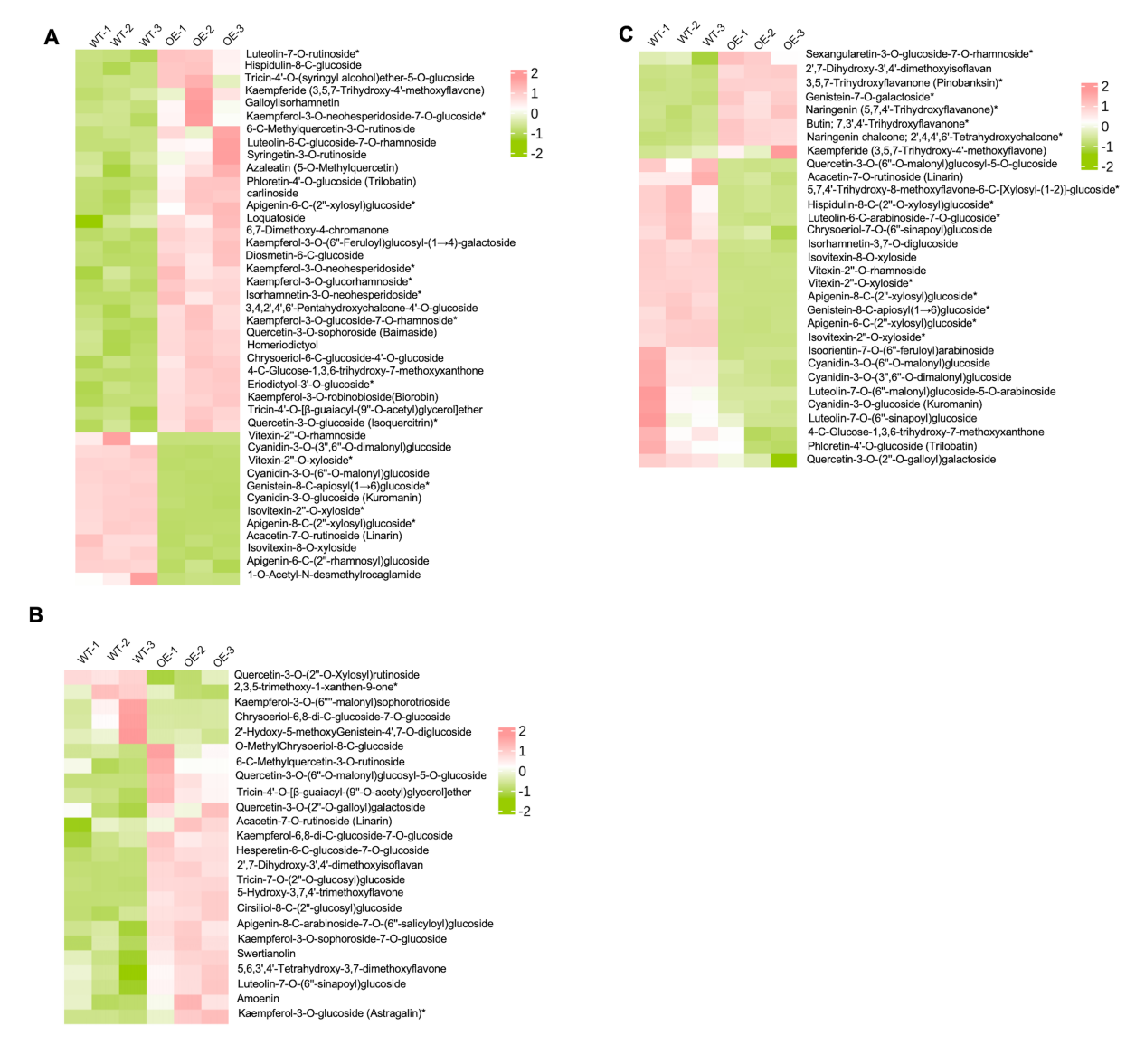


**Supplementary Figure 19. *SiTAF10* promotes flavonoid accumulation** **in foxtail millet leaves. (A-C)** Differentially accumulated flavonoid metabolites in leaves of *SiTAF10*-OE and ‘Ci846’ (WT) plants under normal **(A)**, NaCl stress **(B)**, and drought **(C)** conditions. Data represent mean ± SD of three biological replicates. Statistical significance was determined by Student’s *t*-test (P < 0.05).


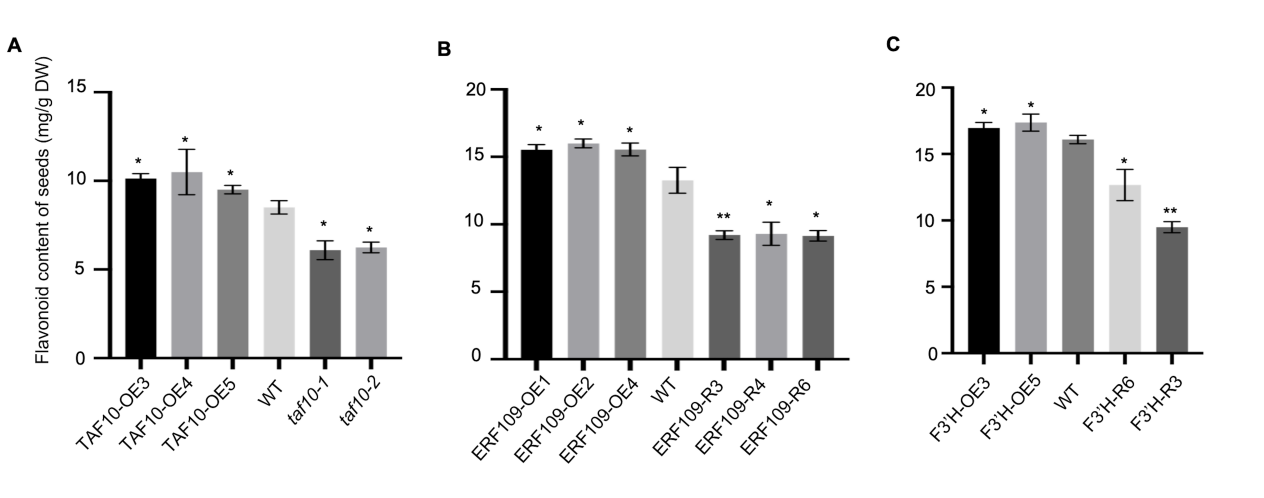


**Supplementary Figure 20. *SiTAF10*, *SiERF109*, *SiF3'H* positively regulate flavonoid accumulation in foxtail millet seeds.**

**(A)** Flavonoid content in seeds of WT, *SiTAF10*-overexpression (TAF10-OE), and *taf10* mutant lines. **(B)** Flavonoid content in seeds of WT, *SiERF109*-overexpression (ERF109-OE), and *SiERF109*-RNAi (ERF109-R) lines. **(C)** Flavonoid content in seeds of WT, *SiF3'H*-overexpression (F3'H-OE), and *SiF3'H*-RNAi (F3'H-R) lines. Data represent mean ± SD of three biological replicates. Statistical significance was determined by Student's *t*-test (*P < 0.05, **P < 0.01).

**
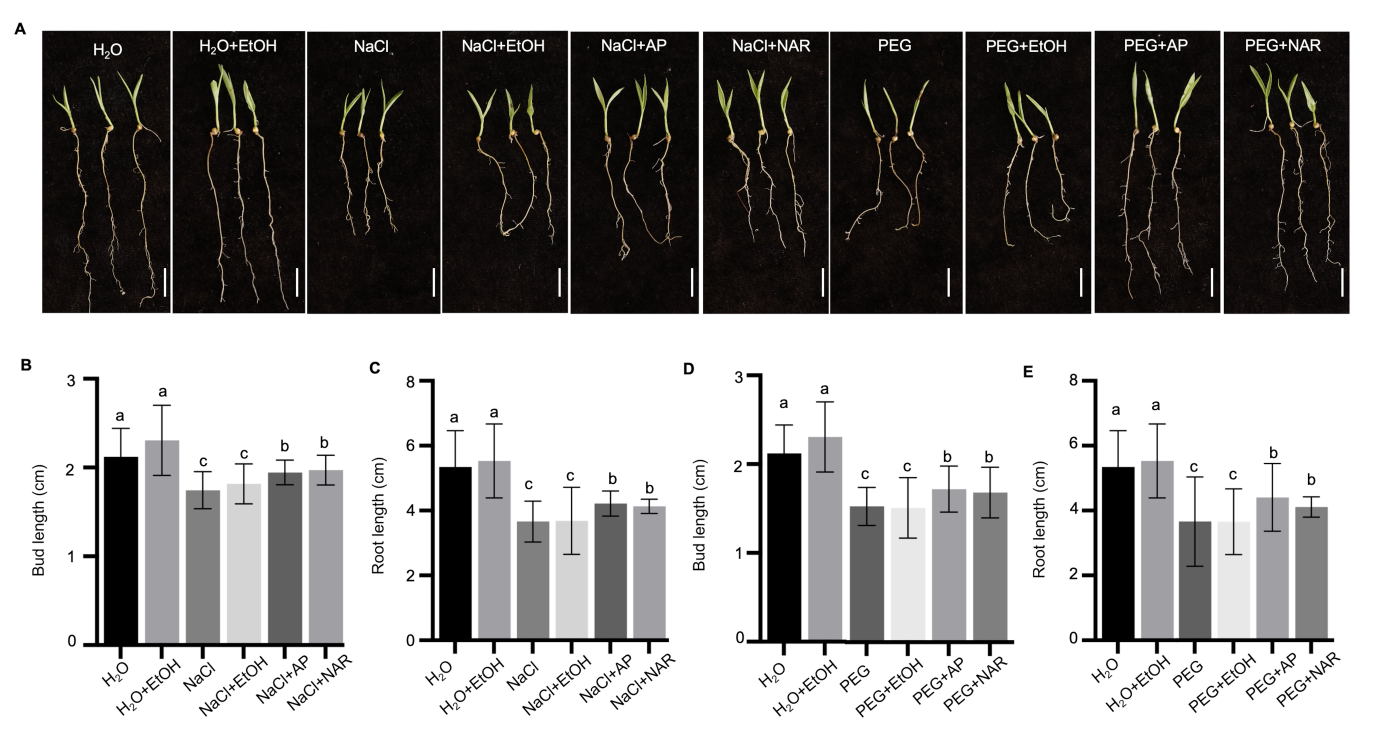
****Supplementary Figure 21.** **Effects of solute (anhydrous ethanol) on the growth of foxtail** **millet seedlings.**

**(A)** Phenotypic comparison of foxtail millet seedlings grown under H_2_O, H_2_O + EtOH, 175 mM NaCl, 175 mM NaCl+ EtOH, 175 mM NaCl + apigenin (AP; 1 μM), 175 mM NaCl + naringenin (NAR; 2 μM), 10% PEG, 10% PEG+ EtOH, 10% PEG + 1 μM AP, 10% PEG + 2 μM NAR. Scale bars = 1 cm. **(B-E)** Bud length **(B** and **D)** and root length **(C** and **E)** of foxtail millet seedings under conditions described in A respectively. Data represent mean ± SD (n = 3 biological replicates). Different letters indicate significant differences (P < 0.05, Tukey's test).


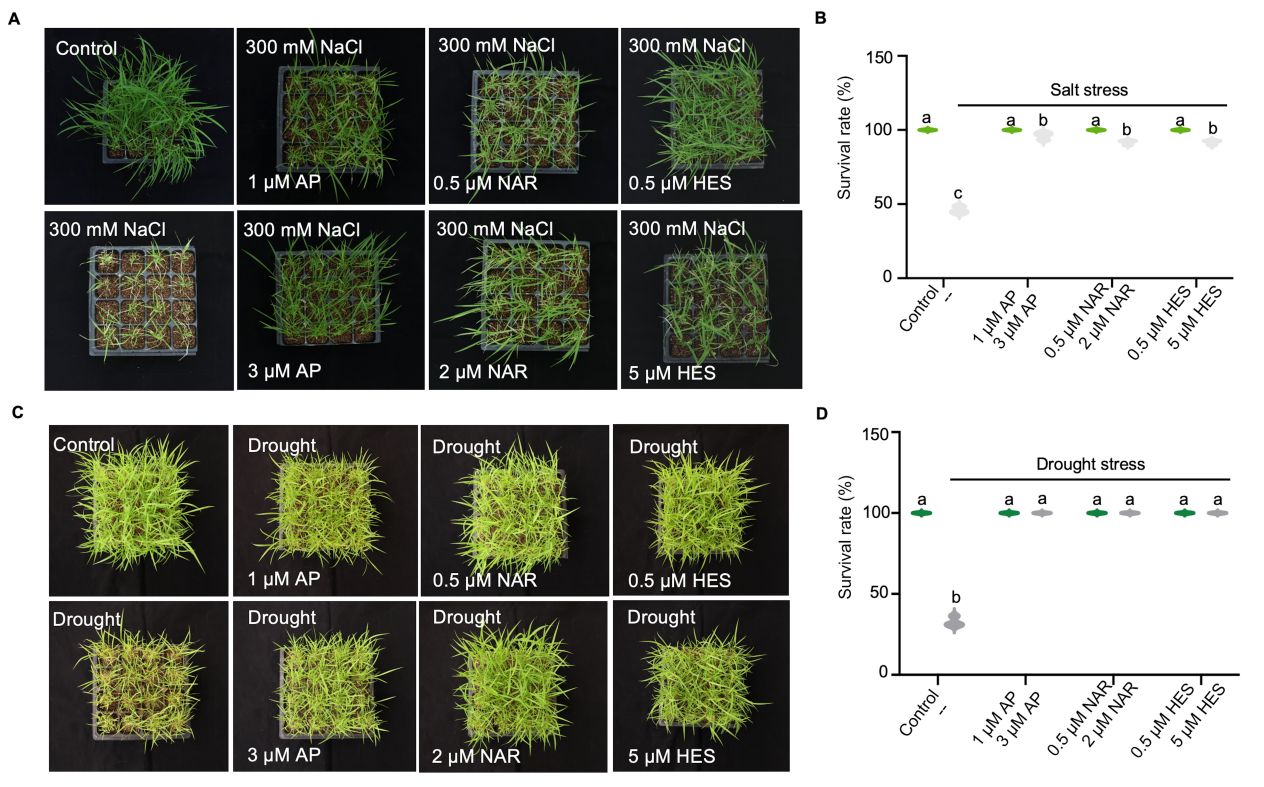


**Supplementary Figure 22. Effects of flavonoids on abiotic stress tolerance in foxtail millet seedlings.**

**(A)** Phenotypic comparison of 14-day-old seedlings after 7-day treatment under salt stress (300 mM NaCl) alone, or with supplementation of apigenin (AP; 0.5 μM or 1 μM), hesperetin (HES; 0.5 μM or 5 μM), or naringenin (NAR; 0.5 μM or 2 μM). **(B)** Corresponding survival rates of seedlings in **(A)**. Data represent mean ± SD (n = 3 biological replicates). Different letters indicate significant differences (P < 0.05, Tukey's test). **(C)** Phenotypic comparison after 14-day treatment of drought alone, or with supplementation of AP, HES, or NAR (concentrations as in A). **(D)** Corresponding survival rates of seedlings in **(C)**. Statistical analysis is same as **(B)**.


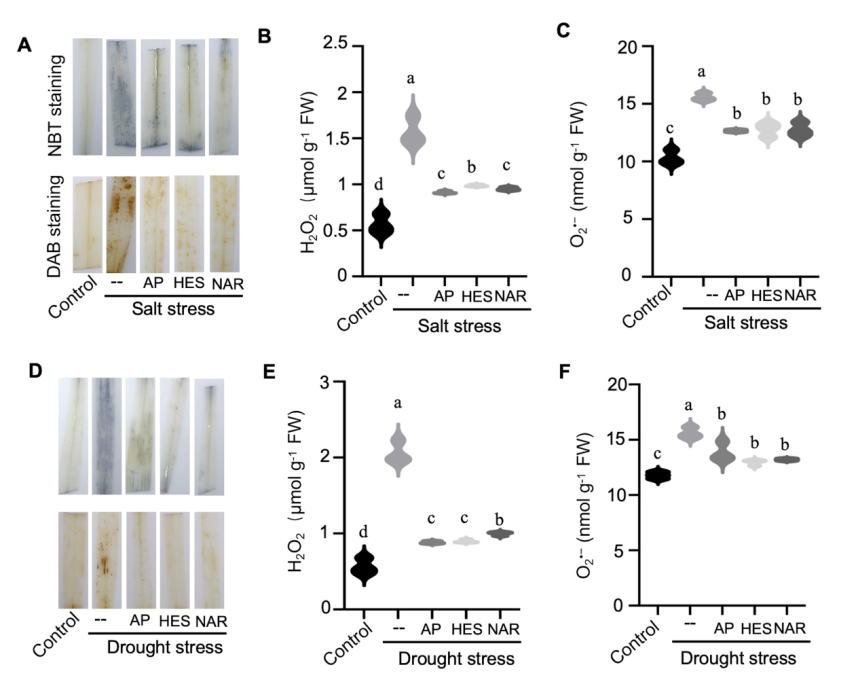


**Supplementary Figure 23.** **ROS detection in flavonoid-treated foxtail millet plants.**

**(A)** NBT staining of leaves showing O_2_^•−^ accumulation in untreated control, salt-stressed (300 mM NaCl) seedlings, and salt-stressed seedlings treated with AP, HES, or NAR. **(B** and **C)** H_2_O_2_ and O_2_^•−^ levels of seedlings from **(A)**. Data represent mean ± SD (n = 3 biological replicates). Different letters indicate significant differences (P < 0.05, Tukey's test). **(D)** DAB staining of leaves showing H_2_O_2_ accumulation under normal conditions, drought stress, and drought stress with AP, HES, or NAR treatment. Staining was performed on leaves from 14-day-old 'Ci846' seedlings corresponding to the phenotypes shown in **Figure 6A** and **6C**. Flavonoid concentrations were as described in **Supplemental Figure 22**. **(E** and **F)** H_2_O_2_ and O_2_^•−^ levels of seedlings from **(D)**. Data represent mean ± SD (n = 3 biological replicates). Different letters indicate significant differences (P < 0.05, Tukey's test).


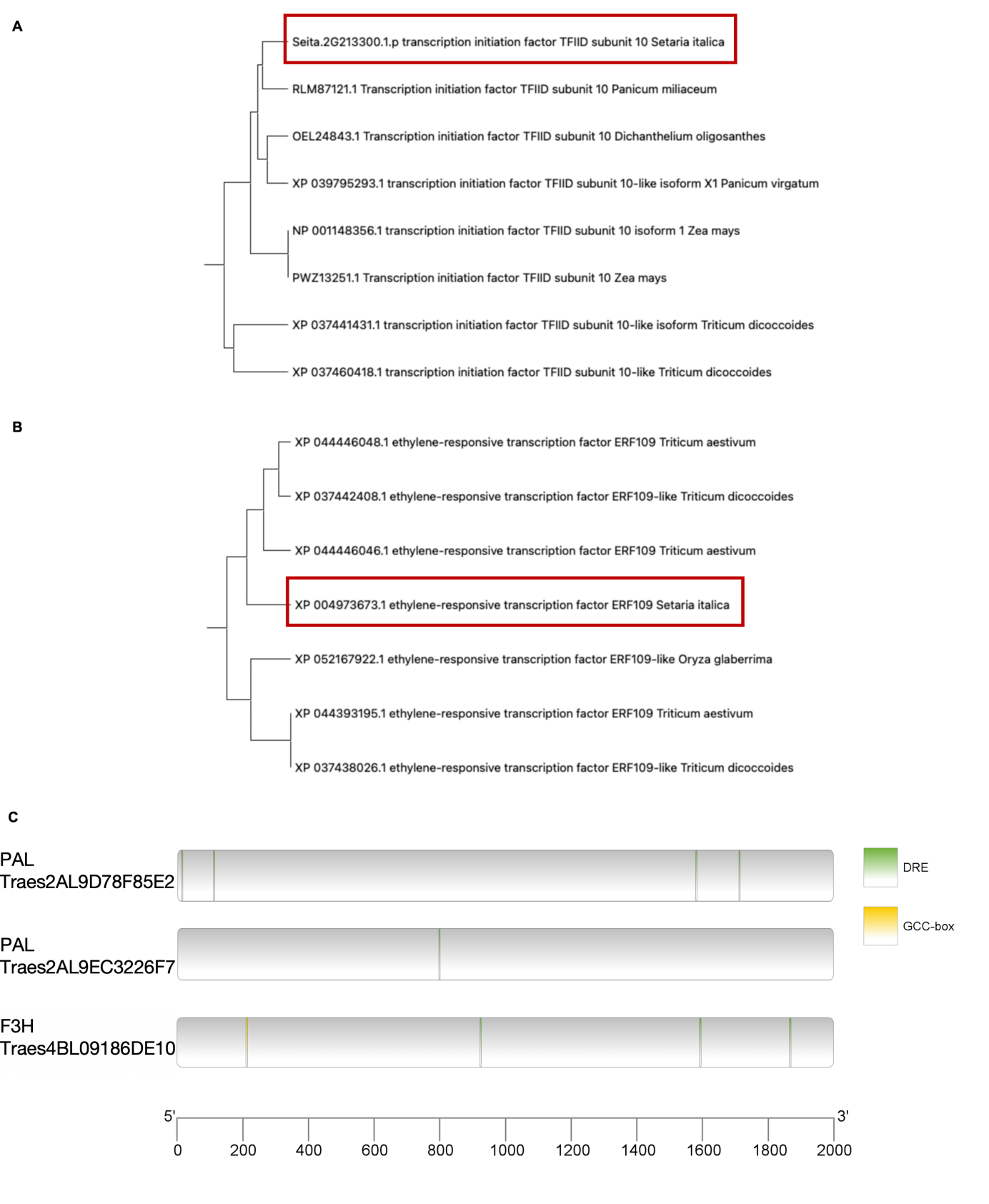


**Supplementary Figure 24. Evolutionary tree analysis of TAF10 and ERF109 and analysis of cis-acting regulatory elements in the wheat *TaPALs* and *TaF3H* promoters*.***

**(A)** Evolutionary tree analysis of TAF10. **(B)** Evolutionary tree analysis of ERF109. **(C)** Schematic representation of the 2,000 bp promoter region of *SiPAL* and *SiF3H*, highlighting predicted cis-regulatory elements. Key elements identified include: GCC-boxes and Dehydration-responsive elements (DRE core).
